# The complexity of outcome measure selection within multiple long-term condition research: an analysis of exercise-based rehabilitation trials

**DOI:** 10.1186/s13063-025-09363-y

**Published:** 2025-12-13

**Authors:** James R. Manifield, Rachael A. Evans, Susan M. Smith, Anne E. Holland, Søren T. Skou, Rod S. Taylor, Sally J. Singh

**Affiliations:** 1 Respiratory Sciences, University of Leicester, Leicester, UK; 2 NIHR Biomedical Research Centre – Respiratory, University Hospitals of Leicester NHS Trust, Leicester, UK; 3 Discipline of Public Health and Primary Care, Trinity College Dublin, Dublin, Ireland; 4 Department of Physiotherapy, Alfred Health, Melbourne, Australia; 5 School of Translational Medicine, Monash University, Melbourne, Australia; 6 Center for Muscle and Joint Health, Department of Sports Science and Clinical Biomechanics, University of Southern Denmark, Odense, Denmark; 7 The Research and Implementation Unit PROgrez, Department of Physiotherapy and Occupational Therapy, Næstved-Slagelse-Ringsted Hospitals, Slagelse, Denmark; 8 MRC/CSO Social and Public Health Sciences Unit, University of Glasgow, Glasgow, UK; 9 Robertson Centre for Biostatistics, University of Glasgow, Glasgow, UK

**Keywords:** Multimorbidity, Rehabilitation, Exercise

## Abstract

**Background:**

Consistent evidence has demonstrated the health benefits of exercise-based rehabilitation across various long-term conditions (LTC); however, evidence in people with multiple LTC (MLTC; or ‘multimorbidity’) is unclear. Development and evaluation of these interventions for MLTC requires a consensus on relevant outcome measures. We aimed to map the outcomes collected and reported in trials of exercise-based rehabilitation in people with MLTC to an existing core outcome set for MLTC (COSmm), and compare to outcomes reported in exercise-based rehabilitation trials of single LTC.

**Methods:**

A secondary analysis of two systematic reviews assessing exercise-based rehabilitation in MLTC. Outcome measures within included trials were categorised into domains and mapped to the existing COSmm. Domains and measures were also extracted from an overview of systematic reviews investigating exercise-based rehabilitation across single LTC.

**Results:**

Two hundred seventeen outcome measures across 59 trials of exercise-based rehabilitation for MLTC were identified. The most common domains were exercise capacity (26 outcomes across 41 trials (69.5%)) and health-related quality of life (18 outcomes across 35 trials (59.3%)). Mapping to COSmm showed consistency in the collection and reporting of outcomes in the domains of physical function, health-related quality of life, mental health, and intervention adherence. However, no outcome measures could be mapped to domains of communication, shared decision-making, prioritisation, or quality healthcare. Analysis of the overview of systematic reviews across single LTC found heterogeneity in outcome measures with common measures being relatively similar to those reported in MLTC populations.

**Conclusion:**

Current outcomes collected and reported in published trials of exercise-based rehabilitation for MLTC appear to only partially map to the domains of the COSmm. This review highlights the need to consider the specific core outcome set domains needed for collection in future trials of intervention for MLTC including exercise-based rehabilitation.

**Supplementary Information:**

The online version contains supplementary material available at 10.1186/s13063-025-09363-y.

## Background

The co-existence of two or more long-term conditions (LTC; multiple LTC (MLTC) or ‘multimorbidity’) [1] is associated with increased risk of disability and mortality, as well as reduced health-related quality of life (HRQoL) and higher healthcare costs [2]. Furthermore, symptom burden is magnified and complicated when individuals present with more than one LTC, with common symptoms across LTCs including dyspnoea, pain, anxiety, and depression [3].

Currently, exercise-based rehabilitation services centre around single diseases, largely focusing on cardiac and pulmonary disease, although evidence from an overview of systematic reviews exists for the benefits of exercise across 25 single LTCs [4]. These interventions for specific conditions are not designed to meet the more complex needs of those with MLTC. A shift to a more personalised MLTC focused approach has been suggested by various commentators [5, 6] to address the limitations associated with disease-specific programmes. Initial literature on exercise-based rehabilitation for individuals living with MLTC suggests that these interventions are feasible [7, 8], safe [9], and may improve exercise capacity and HRQoL in this population [9–11].

Among the immediate challenges of assessing newly developed interventions is the selection of outcomes and outcome measures. Commonly, key outcomes are defined by development of core outcome sets (COS)—an agreed standardised set of outcomes that should be measured as a minimum [12]—which are developed using a formal consensus-based approach, and guided by an internationally agreed framework. In the field of exercise-based rehabilitation and/or exercise-based training, COS have been proposed for pulmonary rehabilitation in chronic obstructive pulmonary disease (COPD) [13], and are under development for cardiac rehabilitation [14].

For research involving people with MLTC, a Delphi panel reached consensus on 17 outcome domains for inclusion in a COS (COS for multimorbidity research (COSmm)) with the highest rated being HRQoL, mental health, and mortality [15]. A wide range of potential outcome measures that could be used to assess these domains were included following Round 1 of the Delphi process, indicating that consensus for specific measures would be challenging and beyond the scope of the COSmm. The developers of the COSmm recognised that it would not be practical or appropriate to include all 17 core outcomes in studies of MLTC and recommended that outcomes should be based on individual study aims and interventions. Furthermore, the COSmm was not specifically developed for exercise-based rehabilitation research. The development of a COS for exercise-based rehabilitation would allow the effectiveness of these programmes and expected mechanisms of action to be evaluated in a population with MLTC, via outcome measures that are relevant for a wide range of LTC. Consistency in collection and reporting of outcome measures will, therefore, allow for direct comparisons across future trials and enable evidence syntheses to determine the efficacy of exercise-based rehabilitation interventions within this population.

The aims of this study were (i) to map the reported outcome measures of exercise-based rehabilitation intervention trials undertaken in MLTC against the domains recommended by the COSmm [15], and (ii) to compare outcome measures reported within exercise-based rehabilitation research for those with MLTC and those with single LTC.

## Methods

Given the availability of two recently published systematic reviews investigating the effects of exercise-based rehabilitation for people with MLTC [9, 10], outcome measures were extracted from included studies across both reviews. The inclusion criteria for these reviews are summarised in Table 1.

**Table 1:** Study inclusion criteria within systematic reviews of exercise-based rehabilitation for people with multiple long-term conditions

	**Bricca et al. **[9]	**Barker et al. **[10]
Design	Randomised controlled trials (RCTs)	RCTs, non-RCTs, and cohort studies
Participants	RCTs reporting at least 80% of adults (> 18 years) with ≥ 2 of the following conditions: osteoarthritis of the knee or hip, heart failure, ischemic heart disease, hypertension, type 2 diabetes mellitus, chronic obstructive pulmonary disease and depression	Participants with ≥ 2 chronic disease defined by the World Health Organisation (WHO) as ‘health problems that require ongoing management over a period of years or decades’
Intervention	Exercise therapy (defined as ‘a regimen or plan of physical activities designed and prescribed for specific therapeutic goals with the purpose of restoring normal physical function or to reduce symptoms caused by diseases or injuries’) interventions with or without additional pharmacotherapy or other adjuvant interventions	Rehabilitation programmes of at least 4 weeks duration that included exercise with or without any form of education or psychological support, delivered in any setting
Comparison	Usual care (e.g. counselling from their health care provider), at the time the trial was conducted, and comparator groups non-exposed such as wait-and-see and placebo treatments	Usual medical care or other interventions that excluded exercise training
Outcomes	Studies that assessed at least one of the following outcomes: Physical health (including objectively measured and self-reported physical function) Psychosocial health (including health-related quality of life (HRQoL), depression and anxiety symptoms) Adverse events The rationale for including these outcomes is based on a consensus study which identified outcomes for multimorbidity intervention studies and the fact that they are generic and widely used across the conditions of interest	Exercise capacity (measured by 1 or more of laboratory-based exercise testing and/or field-based walking tests) Secondary outcomes included: HRQoL, activities of daily living, cardiometabolic outcomes, lipid profiles, body mass index (BMI), mental health outcomes, symptom scores, resource utilization, health behaviours, economic outcomes, and adverse events

Extracted outcome measures (specific instruments (e.g. questionnaires, physical tests) used to assess outcomes) were organised into domains (i.e. high-level categories that grouped outcome measures which shared similar themes) following discussions with senior authors (SJS, RT, RE) who have expertise in the field of exercise-based rehabilitation. A pragmatic approach was followed, whereby similar measures (e.g. VO_2_ peak and VO_2_ maximum; heart rate peak and heart rate maximum), along with the same measures defined in different ways across studies (e.g. sit-to-stand and five times sit-to-stand) were grouped together.

Outcome measures were mapped against the previously developed COSmm [15]. For this work, the COSmm domains of ‘HRQoL’ and ‘self-rated health’ were combined. Furthermore, due to the closely related concepts of ‘exercise capacity’ and ‘physical function’, outcome measures from exercise-based intervention studies that the authors would consider measures of ‘exercise capacity’ were classified as ‘physical function’ to encompass both capacity and performance when mapping to the broader COSmm.

Outcome measures from exercise-based rehabilitation in MLTC populations were compared to those reported across single LTC research from a previous overview of systematic reviews, the full methods of which are detailed elsewhere [4]. In brief, the inclusion criteria included systematic reviews investigating the efficiency of exercise-based rehabilitation compared with usual care, control or alternative non-exercise interventions in adults diagnosed with at least one LTC. The outcomes of interest included clinical events, exercise capacity (aerobic capacity and/or strength), frailty, health-related quality of life (disease specific and generic measures), disability, and physical activity. Three LTC were included in more than one review (cancer: solid tumour, haematological and advanced metastatic; arthritis: hip osteoarthritis, knee osteoarthritis and rheumatoid arthritis; and painful condition: chronic low back pain and fibromyalgia) to reflect disease subtypes. Data relating to outcome measures (both pooled and non-pooled) were extracted by JM from the 25 LTC which showed effectiveness of exercise-based rehabilitation [4]. Radial plots were created using the data visualisation software Flourish (https://flourish.studio).

## Results

### Multiple long-term conditions

**Fig. 1:**
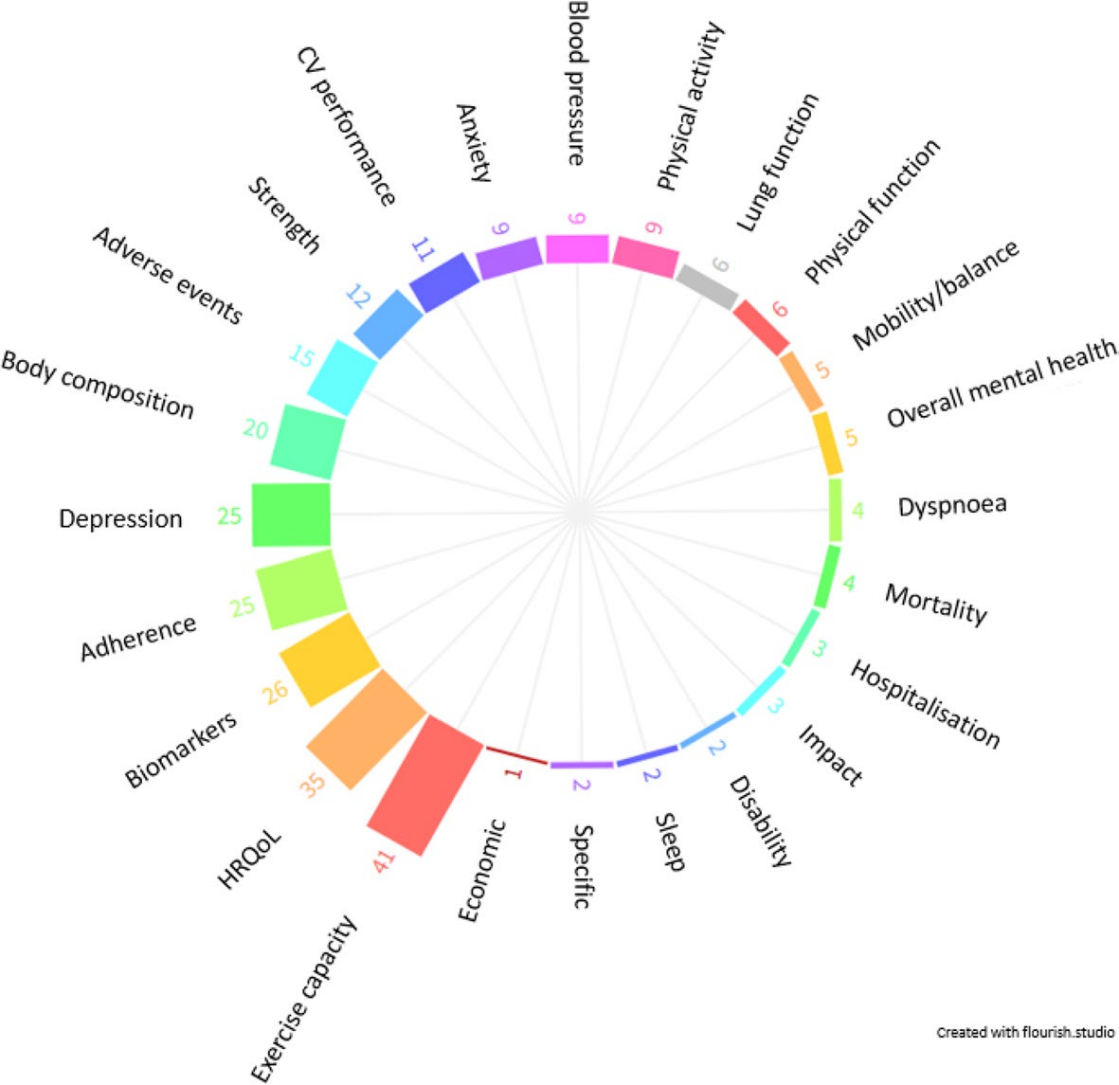
Outcome domains organised from extracted outcome measures across multiple long-term conditions (MLTC). Note: numbers represent total number of studies (out of 59) reporting each outcome domain. Abbreviations: HRQOL, health-related quality of life; CV, cardiovascular

Two hundred seventeen outcome measures were extracted from 59 individual studies (60 reports) investigating exercise-based rehabilitation interventions for those with MLTC, included within the 2 MLTC systematic reviews [9, 10]. These were organised into 24 domains, the most common of which were exercise capacity (41 studies (69.5%)) and HRQoL (35 studies (59.3%); Fig. 1). A list of all reported outcome domains and measures are presented in Table S1, and a list of abbreviations are reported in Table S2.

#### Exercise capacity

**Fig. 2:**
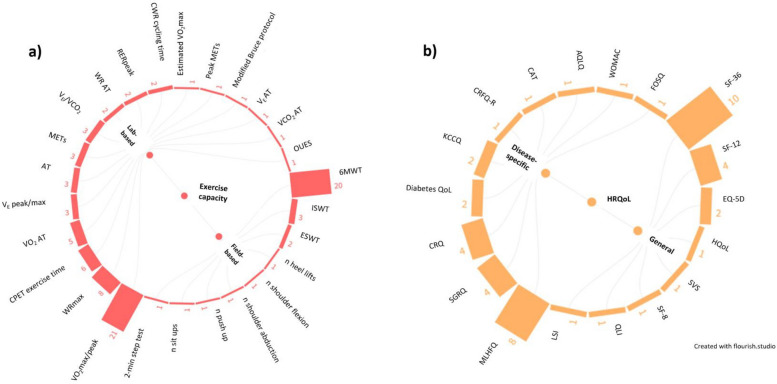
Outcome measures reported within exercise-rehabilitation trials for multiple long-term conditions, organised into **a** exercise capacity (across 41 studies) and **b** health-related quality of life (across 35 studies) domains. Note: numbers represent total number of studies. Abbreviations: 6MWT, 6-min walk test; AQLQ, asthma quality of life questionnaire; AT, anaerobic threshold; CAT, chronic obstructive pulmonary disease (COPD) assessment questionnaire; CFQ-R, cystic fibrosis questionnaire-revised; CRQ, chronic respiratory disease questionnaire; CPET, cardio-pulmonary exercise test; CWR, constant work rate; EQ-5D, EuroQol-5-dimensions; ESWT, endurance shuttle walk test; FOSQ, functional outcomes of sleep questionnaire; HQOL, Hacepette quality of life; ISWT, incremental shuttle walk test; KCCQ, Kansas city cardiomyopathy questionnaire; LSI, life satisfaction index; METS, metabolic equivalent of tasks; MLHFQ, Minnesota living with heart failure questionnaire; OUES, oxygen uptake efficiency slope; QLI, quality of life index; QoL, quality of life; RER, respiratory exchange ratio; SF-(8,12,36), (8, 12, 36)-item short -item short form survey; SGRQ, St. George’s respiratory questionnaire; SVS, subjective vitality scale; VCO_2_, volume of carbon dioxide production; V_E_, minute ventilation; VO_2_, volume of oxygen consumption; WOMAC, Western Ontario and McMaster Universities osteoarthritis index

Twenty-six outcome measures relating to exercise capacity were reported (Fig. 2a; Table S1) consisting of 17 lab-based and 9 field-based measures, reported in 25 (42.4%) and 23 (39.0%) studies, respectfully. Both lab- and field-based outcome measures of exercise capacity were reported in 7 (11.9%) studies. For lab-based measures, the most common was maximal or peak oxygen uptake (VO_2_ max/peak; 21 studies (35.6%)), and for field-based measures, the 6-min walk test distance (6MWT) was the most common (20 studies (33.9%)).

#### Health-related quality of life

Eighteen outcome measures relating to HRQoL were reported across studies (Fig. 2b; Table S1), and consisted of 8 general (reported in 19/59 (32.2%) studies) and 10 disease-specific (reported in 23/59 (39.0%) studies) measures. Both general and disease specific HRQoL outcome measures were used in 7 (11.9%) studies. The 36-Item Short Form Survey (SF-36) was the most commonly used outcome measure (10 (16.9%) studies).

#### Mapping to the core outcome set for multimorbidity (COSmm)

**Fig. 3:**
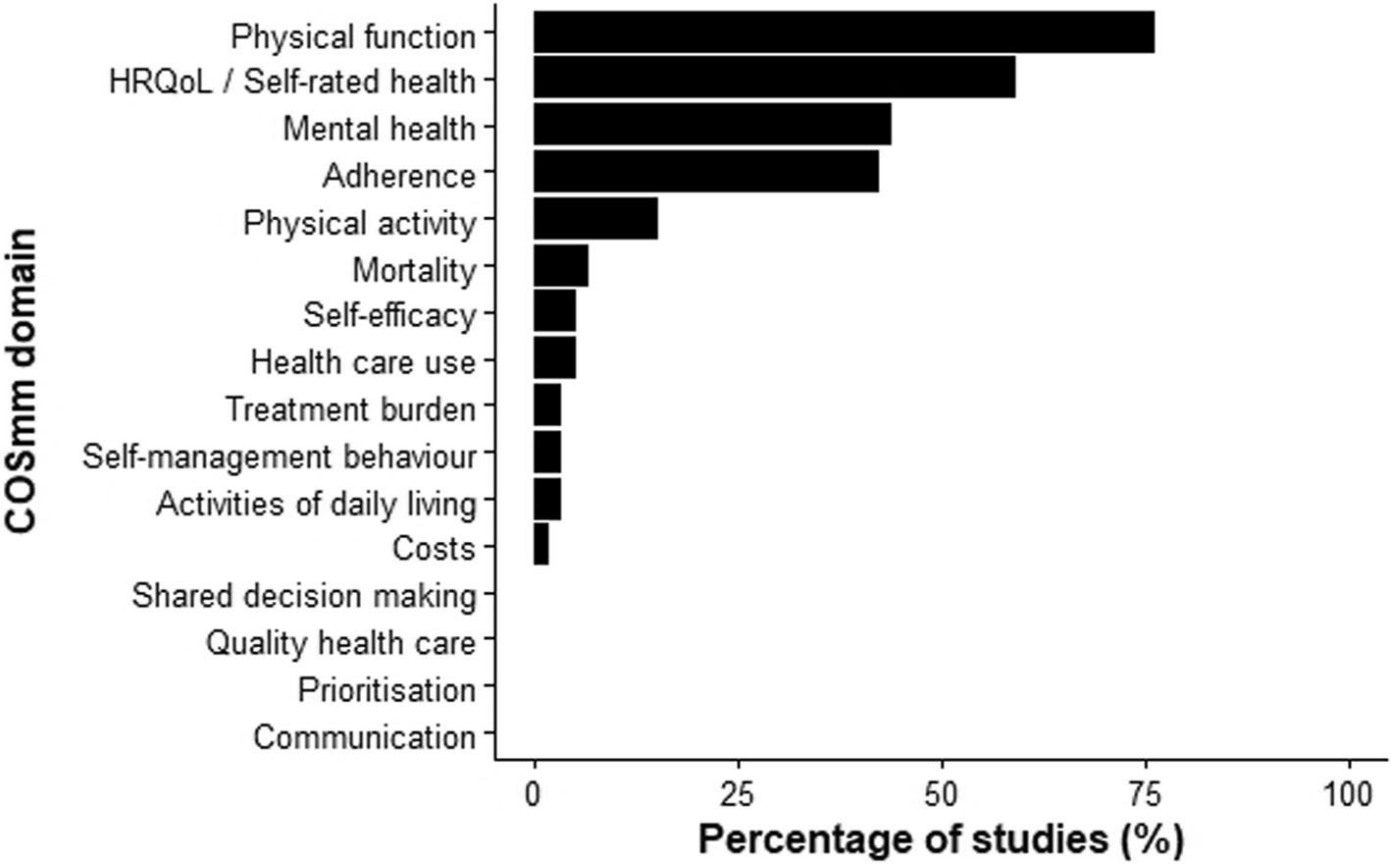
The mapping of reported outcomes used within exercise-based rehabilitation studies for multiple long-term conditions (*n* = 59) against domains suggested in the core outcome set for multimorbidity research (COSmm) [15]. Abbreviations: HRQoL, health-related quality of life

When mapped against the domains from the COSmm [15], those most commonly reported across the 59 exercise-based rehabilitation intervention studies for MLTC populations were physical function (*n* = 45; 76.3%), HRQoL/self-rated health (*n* = 35; 59.3%), mental health (*n* = 26; 44.1%), and adherence (*n* = 25; 42.4%; Fig. 3). No studies reported outcome measures that could be categorised into the communication, shared decision-making, prioritisation, or quality healthcare COSmm domains.

Specific outcome measures that were mapped against the COSmm domains are reported in Table S3. Additional outcome measures reported in exercise-based intervention studies for MLTC could not be classified into the domains of the COSmm, and mainly related to body composition, biomarkers, blood pressure, and lung function measures.

### Single long-term conditions

Using the overview of systematic reviews that explored single LTC exercise rehabilitation trials [4], 722 outcome measures were extracted from 30 systematic reviews (comprising of 1049 studies) across 25 individual LTC, and subsequently organised into 29 domains (Table S4). In line with outcome domains reported within MLTC trials, the most common across single LTC were exercise capacity (22 LTC (88%); 23 reviews (560 studies)), and HRQoL (22 LTCs (88%); 27 reviews (482 studies)).

#### Exercise capacity

Fifty-eight outcome measures for assessing exercise capacity were reported (Fig. S1a), of which, 34 were field-based measures (reported across 19 LTCs (332 studies)), and 24 were lab-based measures (reported across 17 LTCs (228 studies)). The most commonly used measures were similar to those reported within MLTC trials, namely VO_2_ max/peak (17 LTCs, 98 studies) and the 6MWT (16 LTCs (152 studies)).

#### Health-related quality of life

Sixty-two outcome measures for assessing HRQoL were reported (Fig. S1b), of which 42 were disease-specific (67.7%; reported across 21 LTCs (229 studies)) and 20 were general (32.3%; reported across 19 LTCs (253 studies)) measures of HRQoL. Both disease-specific and general outcome measures were reported across 19 LTC. As in MLTC trials, the SF-36 was the most commonly used outcome measure for HRQoL across LTC (18 LTC (130 studies)).

#### Other outcome domains

The top 3 most commonly used outcome measures for each domain are presented in Table 2. Twenty-four domains (82.8%) were used in both single LTC and MLTC research. Similar commonly used outcome measures across individual LTC and within MLTC were observed in 12 domains (exercise capacity, HRQoL, strength, biomarkers, body composition, depression, anxiety, adverse events, physical activity, blood pressure, cardiovascular performance, and hospitalisation). A list of all reported outcome measures within exercise-based rehabilitation across single LTC are presented in Table S4.

**Table 2:**
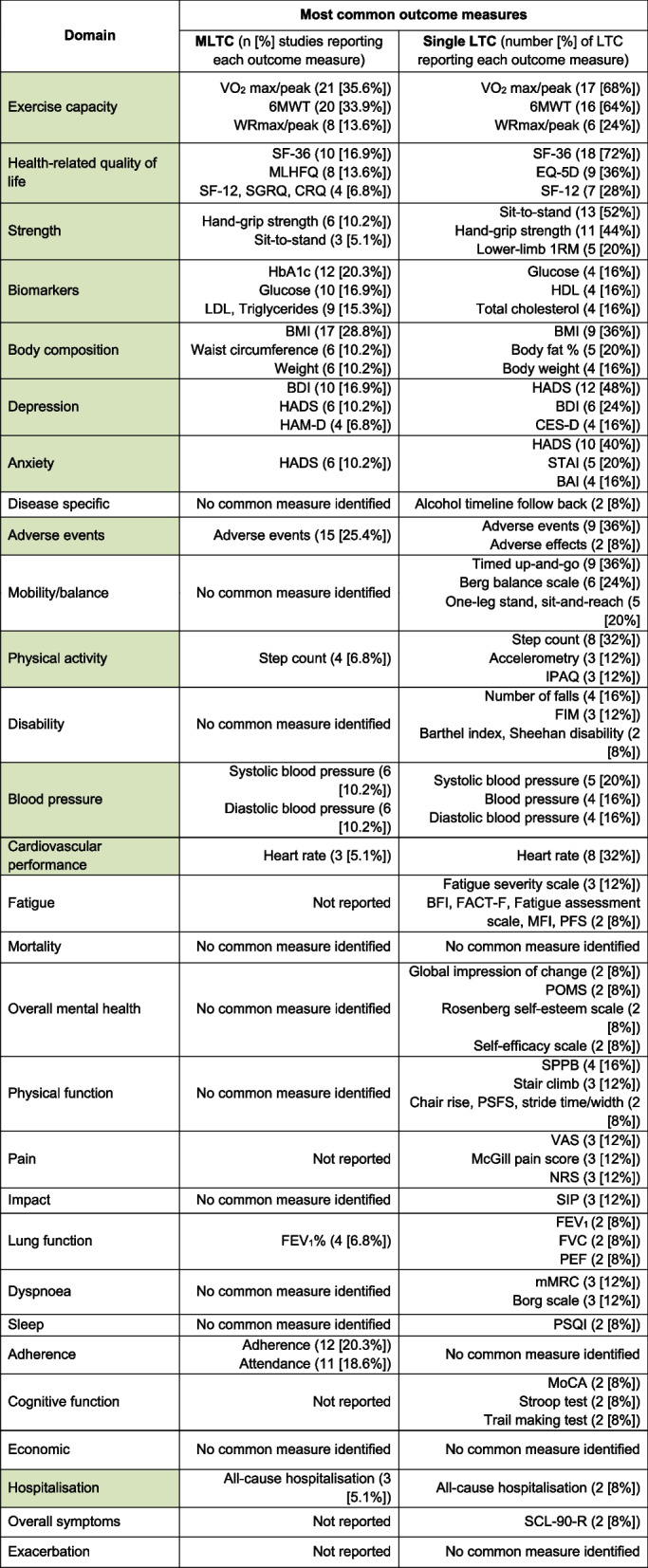
The top 3 most common outcome measures reported in exercise-based rehabilitation intervention research within studies including MLTC and across systematic reviews for single LTC

*1RM *one-repetition maximum, *6MWT *six-minute walk test, *BAI *Beck anxiety index, *BDI *Beck depression inventory, *BFI *brief fatigue inventory, *BMI *body mass index, *CES-D *centre for epidemiological studies depression scale, *CRQ *chronic respiratory disease questionnaire, *EQ-5D *EuroQol-5-dimensions, *FACT-F *functional assessment of cancer therapy-fatigue, *FEV*_1 _forced expiratory volume in one second, *FIM *functional independence measure, *FVC *forced vital capacity, *HADS *hospital anxiety and depression scale, *HAM-D *Hamilton depression rating scale, *HbA1c *Haemoglobin A1c, *HDL *high-density lipoprotein, *IPAQ *international physical activity questionnaire, *LDL *low-density lipoprotein, *MFI *multidimensional fatigue inventory, *MLHFQ *Minnesota living with heart failure questionnaire, *mMRC *modified medical research council dyspnoea scale, *MoCA *Montreal cognitive assessment, *NRS *numeric rating scale, *PEF *peak expiratory flow, *PFS *piper fatigue scale, *POMS *profile of mood states, *PSFS *patient specific functional scale, *PSQI *Pittsburgh sleep quality index, *SCL-90-R *Symptom Checklist-90-Revised, *SF-12/36 *12/36-item short form survey, *SGRQ *St. George’s respiratory questionnaire, *SIP *sickness impact profile, *SPPB *short physical performance battery, *STAI *state-trait anxiety index, *VAS *visual analogue scale, *VO*_2 _volume of oxygen consumption, *WR *work rate.

Percentages are expressed out of 59 studies for multiple long-term conditions (MLTC), and out of 25 single long-term conditions (LTC). ‘No common measure identified’ was defined as the most common outcome measure within a particular domain being reported across <5% of MLTC studies or LTC. Green highlighted domains represent similarities in common outcome measures between single- and MLTC research.

## Discussion

### Summary of main findings

Exercise is acknowledged as a valuable intervention in a wide range of single LTC and of potential value in those with MLTC. Our analysis identifies a large range of outcome domains and measures used within exercise-based rehabilitation intervention research across both MLTC [9, 10] and single LTC [4]. When mapped against the COSmm domains [15], the most commonly reported were physical function (including measures of exercise capacity) (76.3%), HRQoL (59.3%), mental health (44.1%), and intervention adherence (42.4%). However, other COSmm domains including communication, shared decision-making, prioritisation, and quality healthcare were much less commonly reported.

Specific measures used to assess these outcome domains were similar between single LTC and MLTC studies. The more generic (i.e. not disease-specific) measures of HRQoL were the most widely used across individual LTC. Within the MLTC literature, disease-specific measures were still being used, highlighting the challenge of both outcome measure and participant selection within intervention research for MLTC.

### Interpretation of findings

The complex symptom burden within individuals living with MLTC, and therefore wide range of potential outcomes, is likely to translate into assessment burden if researchers choose to measure all recommended outcomes and potential presenting symptoms. The authors of the COSmm caution that including all suggested outcomes within an individual study may result in excessive burden on participants and increase the risk of type 1 errors [15]. Furthermore, reports from both participants and assessors within two pilot RCTs investigating the effects of exercise-based rehabilitation in people with MLTC [16, 17] suggested that assessments were time-consuming, highlighting the need for refining the number and efficiency in choice of outcome measures in this population. This finding, however, is not consistent across all MLTC exercise-based trials, with 95% of participants within a recent feasibility trial reporting that they did not find their assessment of their outcomes too overly burdensome [7].

Whilst physical measures of exercise capacity have been prioritised in exercise-based rehabilitation trials to date, and will remain important to establish the efficacy of physical training programmes, it is also important that researchers capture the range of outcomes that are a priority for individuals living with MLTC, including mental health [15]. Interestingly, although exercise capacity was the most commonly reported domain across both single- (88%) and MLTC (69.5%) exercise-based rehabilitation research, not all trials/reviews measured this outcome.

Whilst authors of the COSmm aimed to reach a consensus on both outcome domains and measures [15], this was not possible due to the very wide range of potential outcome measures identified in the first round of their Delphi process. The highest rated within the HRQoL domain of the COSmm matches the most commonly reported within the present analysis, i.e. the: EQ-5D and SF-36.

An additional core outcome set, specifically for trials of interventions to prevent and treat multimorbidity in adults in low- and middle-income countries (COSMOS), has recently been developed [18]. As with the COSmm, HRQoL was among the highest scoring outcomes. Other agreed outcomes for the treatment of multimorbidity included adherence to treatment, adverse events, and out-of-pocket expenditure. As these COS for MLTC (including the COSmm) have been published within the last 10 years, some trials of exercise-based rehabilitation for this population would not have been able to adhere to recommended outcomes.

An important factor to consider when identifying outcomes to use in MLTC trials is patient selection. This is especially relevant for ‘disease-specific’ measures of HRQoL (e.g. Minnesota Living with Heart Failure Questionnaire or St. George’s Respiratory Questionnaire) or biomarkers (HbA1c or glucose levels). Although generic measures are likely the most appropriate for assessing HRQoL in those with MLTC, 65.7% of the studies assessing this outcome utilised disease-specific measures in the present analysis. This finding was likely driven by studies including ‘comorbid’ participants (i.e. with additional conditions in reference to an index condition) and not those specifically with ‘multimorbidity’ (i.e. no priority given to any single LTC) [19]. Given the current lack of consensus on the definition of multimorbidity within the literature [2, 19, 20], we defined a participant to be ‘multimorbid’, if they were not recruited based on an index condition and also have multiple conditions that impact more than one organ system.

Within the review by Bricca et al. [9], not all studies included 100% of participants with multimorbidity (criteria of ≥ 80%), and the majority of RCTs included those with depression and heart failure. Furthermore, the most common disease combination within Barker et al. [10] was COPD and ‘comorbidities (diagnosis not specified)’. It is, therefore, unsurprising that cardiac and pulmonary disease-specific measures were commonly used across domains.

Findings from the two reviews that included those with MLTC suggest that exercise rehabilitation is beneficial in this population in terms of improving functional exercise capacity and HRQoL [9, 10]. This is in contrast to the findings from a broader systematic review including a wider range of interventions for those living with MLTC [21] that showed no evidence of improvements in HRQoL. The authors concluded that future research for MLTC should consider targeting patient health behaviours such as exercise. Recent trials in this area, such as the UK National Institute for Health Research (NIHR) funded ‘PERFORM’ research programme [8, 22–24] and the ‘MOBILIZE’ trial [7, 11] have utilised generic outcome measures of HRQoL (EQ-5D) and other COSmm recommended outcomes including physical function, treatment burden, adherence, mental health, and health care use.

### Strengths and limitations

This unique analysis has identified a large number of outcome measures that have been used in exercise-based rehabilitation interventions both across a range of single LTC and within MLTC. Although the selected systematic review for each LTC within the overview [4] was based on a pre-determined criteria that included ‘reporting the most outcomes of interest’, other eligible reviews within specific LTC may have reported different outcome measures. These systematic reviews were not identified to specifically identify the range of outcome measures used within each LTC, instead, the present analysis provides a snapshot of the current literature in this field. Differences in patient selection as described above (i.e. ‘multimorbidity’ vs ‘comorbidity’) between MLTC studies will likely have influenced the outcome measures used.

When mapping to the COSmm, certain COSmm domains (e.g. ‘HRQoL’ and ‘self-rated health’) were combined. Furthermore, during the development of the COSmm, the ‘physical function’ domain was considered with a focus on occupational therapy and disability measures. The outcome measures from exercise-based trials that we have mapped to this domain focused predominately on exercise capacity, however, we believe these can also be classified as measures of physical function. For future exercise-based rehabilitation trials in MLTC populations, we would recommend that researchers use measures assessing exercise capacity alongside self-reported measures of physical function alone, as recommended in the COSmm.

### Future directions

Whilst this work represents the first step in identifying outcome domains and measures that may be used in exercise-based rehabilitation for this population, future work should aim to formally develop and validate a core set of outcomes with experts in the field and individuals living with MLTC. Our group has recently published a protocol paper outlining how we plan to address this aim [25]. This will involve the following: (i) using the World Health Organization’s International Classification of Functioning, Disability, and Health (ICF) framework to quantify meaningful concepts within outcome measures identified in the present review; (ii) qualitative studies, to determine patient and caregiver perspectives; (iii) expert surveys, to determine healthcare professional perspectives; (iv) an empirical study, to determine the clinical perspective; and (v) an international conference, to reach a consensus on an ICF core set for exercise rehabilitation in MLTC.

‘Consultation-related’ outcomes recommended within the COSmm (i.e. communication, shared decision-making, and prioritisation) are important factors to consider for people with MLTC, especially relating to polypharmacy or within primary care settings; however, we found that these outcomes were not reported within exercise-based rehabilitation trials in the present analysis. It is important to consider communication, shared decision-making, and prioritisation when designing exercise interventions for clinical populations [26]; however, they are rarely operationalised as outcome measures in this field. Future developmental work is needed to capture these outcomes using validated patient-reported outcome measures in the field of exercise-based rehabilitation for MLTC populations.

## Conclusion

We have shown that a large number of outcome domains and outcome measures have been reported in trials of exercise-based rehabilitation for people with MLTC. These outcome measures appear to only partially map to the domains of the COSmm.

Due to the complex symptom burden of those with MLTC there is, inevitably, a tension between generic and disease-specific outcome measures, and patient burden. If exercise-based interventions have promise in this population, the development of an agreed set of outcomes in crucial.

## Supplementary Information

Supplementary Material 1. Table S1. Outcome domains and measures reported within studies of exercise-based rehabilitation for people with multiple long-term conditions. Table S2. Outcome measure abbreviations. Table S3. Outcome measures reported across exercise-based rehabilitation research in MLTC organised into core outcome set for multimorbidity (COSmm) domains. Figure S1. Outcome measures reported within exercise-rehabilitation research across single long-term conditions (LTC) organised into (a) exercise capacity (across 22 LTC) and (b) health-related quality of life (across 22 LTC) domains Table S4. Outcome domains and measures reported across exercise-based rehabilitation systematic reviews for 25 single long-term conditions (LTC)

## Data Availability

The datasets used and/or analysed during the current study are available from the corresponding author on reasonable request.
