# Supplementary material for: The complexity of outcome measure selection within multiple long-term condition research: an analysis of exercise-based rehabilitation trials
Source: Trials. 2025 Dec 13;27:46. doi: 10.1186/s13063-025-09363-y (PMC12817629; doi:10.1186/s13063-025-09363-y)
Supplement: Supplementary file 1 — Supplementary Material 1. Table S1. Outcome domains and measures reported within studies of exercise-based rehabilitation for people with multiple long-term conditions. Table S2. Outcome measure abbreviations. Table S3. Outcome measures reported across exercise-based rehabilitation research in MLTC organised into core outcome set for multimorbidity (COSmm) domains. Figure S1. Outcome measures reported within exercise-rehabilitation research across single long-term conditions (LTC) organised into (a) exercise capacity (across 22 LTC) and (b) health-related quality of life (across 22 LTC) domains Table S4. Outcome domains and measures reported across exercise-based rehabilitation systematic reviews for 25 single long-term conditions (LTC) [file 13063_2025_9363_MOESM1_ESM.docx]

**Supplementary material**

**Contents**

[**Table S1.** Outcome domains and measures reported within studies of exercise-based rehabilitation for people with multiple long-term conditions. 2](#_Toc190700046)

[**Table S2.** Outcome measure abbreviations. 6](#_Toc190700047)

[**Table S3.** Outcome measures reported across exercise-based rehabilitation research in MLTC organised into core outcome set for multimorbidity (COSmm) domains. 13](#_Toc190700048)

[**Figure S1.** Outcome measures reported within exercise-rehabilitation research across single long-term conditions (LTC) organised into (a) exercise capacity (across 22 LTC) and (b) health-related quality of life (across 22 LTC) domains. 20](#_Toc190700049)

[**Table S4.** Outcome domains and measures reported across exercise-based rehabilitation systematic reviews for 25 single long-term conditions (LTC). 21](#_Toc190700050)

# **Table S1.** Outcome domains and measures reported within studies of exercise-based rehabilitation for people with multiple long-term conditions.

| **Outcome domains** | **Outcome domains (number [%] studies)** |
| --- | --- |
| **Exercise capacity (41 [69.5%])**  *Field-based (23 [39.0%])*  *Lab-based (25 [42.4%])*  *Both (7 [11.9%])** | **Field-based:**  6MWT (20 [33.9%])  ISWT (3 [5.1%])  ESWT (2 [3.4%])  Number of heel lifts (1 [1.7%])  Number of shoulder flexions (1 [1.7%])  Number of shoulder abductions (1 [1.7%])  Number of push ups (1 [1.7%])  Number of sit ups (1 [1.7%])  2MST (1 [1.7%])  **Lab-based:**  VO_2_max/peak (21 [35.6%])  WRmax (8 [13.6%])  CPET exercise time (6 [10.2%])  VO_2_ at AT (5 [8.5%])  V_E_max/peak (3 [5.1%])  AT (3 [5.1%])  METs (3 [5.1%])  V_E_/VCO_2_ (3 [5.1%])  WR at AT (2 [3.4%])  RERpeak (2 [3.4%])  CWR cycling time (2 [3.4%])  Estimated VO_2_max (1 [1.7%])  Peak METs (1 [1.7%])  Modified Bruce protocol (1 [1.7%])  V_E_ at AT (1 [1.7%])  VCO_2_ at AT (1 [1.7%])  OUES (1 [1.7%]) |
| **Health-related quality of life (35 [59.3%])**  *General (19 [32.2%])*  *Disease-specific (23 [49.0%])*  *Both (7 [11.9%])** | **General:**  SF-36 (10 [16.9%])  SF-12 (4 [6.8%])  EQ-5D (2 [3.4%])  SVS (1 [1.7%])  SF-8 (1 [1.7%])  QLI (1 [1.7%])  LSI (1 [1.7%])  HQoL (1 [1.7%])  **Disease specific:**  MLHFQ (8 [13.6%])  SGRQ (4 [6.8%])  CRQ (4 [6.8%])  Diabetes QoL (2 [3.4%])  KCCQ (2 [3.4%])  CFQ-R (1 [1.7%])  CAT (1 [1.7%])  AQLQ (1 [1.7%])  WOMAC (1 [1.7%])  FOSQ (1 [1.7%]) |
| **Biomarkers (26 [44.1%])** | HbA1C (13 [22.0%])  Glucose (10 [16.9%])  LDL (9 [15.3%])  Triglycerides (9 [15.3%])  HDL (8 [13.6%])  Total cholesterol (6 [10.2%])  CRP (5 [8.5%])  IL-6 (5 [8.5%])  Insulin (5 [8.5%])  IL-1 (3 [5.1%])  Lipids (3 [5.1%])  TNF-a (3 [5.1%])  Adiponectin (2 [3.4%])  Cholesterol (2 [3.4%])  GT (2 [3.4%])  HOMA-IR (2 [3.4%])  IL-8 (2 [3.4%])  Leptin (2 [3.4%])  25(OH)D (1 [1.7%])  Albumin (1 [1.7%])  ALP (1 [1.7%])  ALT (1 [1.7%])  AST (1 [1.7%])  BDNF (1 [1.7%])  Beta-thromboglobulin (1 [1.7%])  Blood urine nitrogen (1 [1.7%])  BNP (1 [1.7%])  C-peptide (1 [1.7%])  Cortisol (1 [1.7%])  Creatine (1 [1.7%])  eGFR (1 [1.7%])  IL-10 (1 [1.7%])  IL-12 (1 [1.7%])  IL-13 (1 [1.7%])  IL-2 (1 [1.7%])  IL-4 (1 [1.7%])  IL-5 (1 [1.7%])  NIRS (1 [1.7%])  NT-proBNP (1 [1.7%])  PF4 (1 [1.7%])  PINP (1 [1.7%])  TGF-b (1 [1.7%])  Uric acid (1 [1.7%])  VEGF (1 [1.7%])  YKL-40 (1 [1.7%]) |
| **Adherence (25 [42.4%])** | Adherence (12 [20.3%])  Attendance (11 [18.6%])  Completion (2 [3.4%]) |
| **Depression (25 [42.4%])** | BDI (10 [16.9%])  HADS (6 [10.2%])  HAM-D (4 [6.8%])  PHQ-9 (3 [5.1%])  GDS (2 [3.4%])  ‘depressive symptoms’ (1 [1.7%])  ATQ (1 [1.7%])  BADS (1 [1.7%])  CES-D (1 [1.7%])  DDS17 (1 [1.7%])  SIGHD (1 [1.7%]) |
| **Body composition (20 [33.9%])** | BMI (18 [30.5%])  Waist circumference (6 [10.2%])  Weight (6 [10.2%])  Body fat % (4 [6.8%])  Fat mass (3 [5.1%])  Lean mass (2 [3.4%])  Waist-hip ratio (2 [3.4%])  Fat free mass (1 [1.7%])  Lower limb muscle mass (1 [1.7%])  Neck circumference (1 [1.7%]) |
| **Adverse events (15 [25.4%])** | Adverse events (15 [25.4%]) |
| **Strength (12 [20.3%])** | HGS (6 [10.2%])  Sit-to-stand (4 [6.8%])  Isokinetic quadricep strength (2 [3.4%])  Bicep curl 1RM (1 [1.7%])  Chest press 1RM (1 [1.7%])  Forearm flexion (1 [1.7%])  Knee extension (1 [1.7%])  Knee isometric strength (1 [1.7%])  Lat pulldown 1RM (1 [1.7%])  Leg press 1RM (1 [1.7%])  MEP (1 [1.7%])  MEP % (1 [1.7%])  MIP (1 [1.7%])  MIP % (1 [1.7%])  Peak torque (1 [1.7%])  Quadricep strength (1 [1.7%]) |
| **Cardiovascular performance (11 [18.6%])** | HRmax/peak (5 [8.5%])  HR (3 [5.1%])  HRV (2 [3.4%])  LVEF (2 [3.4%])  HRR (2 [3.4%])  LAVI (1 [1.7%])  LVMI (1 [1.7%])  HR change (1 [1.7%])  HRisowork (1 [1.7%])  Post-exercise HR (1 [1.7%])  SVpeak (1 [1.7%]) |
| **Anxiety (9 [15.3%])** | HADS (6 [10.2%])  BAI (1 [1.7%])  PSS-10 (1 [1.7%])  STAI (1 [1.7%]) |
| **Blood pressure (9 [15.3%])** | DBP (6 [10.2%])  SBP (6 [10.2%])  MAP (2 [3.4%])  Blood pressure (1 [1.7%])  BRS (1 [1.7%])  DBPpeak (1 [1.7%])  FMD (1 [1.7%])  MAPpeak (1 [1.7%])  SBPpeak (1 [1.7%])  Change in blood pressure during exercise (1 [1.7%]) |
| **Physical activity (9 [15.3%])** | Step count (4 [6.8%])  IPAQ (2 [3.4%])  Accelerometry (1 [1.7%])  CHAMPS (1 [1.7%])  Exercise self-efficacy (1 [1.7%])  Godin PA (1 [1.7%])  Exercise min/week (1 [1.7%])  MVPA (1 [1.7%])  PASE (1 [1.7%])  Total EE (1 [1.7%])  Unspecified PA questionnaire (1 [1.7%]) |
| **Lung function (6 [10.2%])** | FEV_1_ % (4 [6.8%])  FEV_1_ (2 [3.4%])  FEV_1_/FVC (2 [3.4%])  FVC (2 [3.4%])  FVC % (2 [3.4%])  ERV (1 [1.7%])  FE_NO_ (1 [1.7%])  PaCO_2_ (1 [1.7%])  PaO_2_ (1 [1.7%])  SaO_2_ (1 [1.7%])  TLC (1 [1.7%])  TLCO % (1 [1.7%]) |
| **Physical function (6 [10.2%])** | 10 m gait speed(1 [1.7%])  Comfortable gait speed (1 [1.7%])  CS-PFP10 (1 [1.7%])  DASI (1 [1.7%])  FPI (1 [1.7%])  MPPB (1 [1.7%])  SPPB (1 [1.7%]) |
| **Mobility/balance (5 [8.5%])** | Sit-and-reach (2 [3.4%])  TUG (2 [3.4%])  Body sway (1 [1.7%])  Functional reach (1 [1.7%])  Tandem stand (1 [1.7%])  Tandem walk (1 [1.7%]) |
| **Overall mental health (5 [8.5%])** | Self-efficacy for exercise (2 [3.4%])  EPQ (1 [1.7%])  POMS (1 [1.7%])  Tai chi exercise self-efficacy (1 [1.7%]) |
| **Dyspnoea (4 [6.8%])** | MRC (2 [3.4%])  mMRC (2 [3.4%]) |
| **Mortality (4 [6.8%])** | All-cause mortality (2 [3.4%])  Cardiovascular mortality (2 [3.4%])  Mortality (2 [3.4%])  Heart failure mortality (1 [1.7%]) |
| **Hospitalisation (3 [5.1%])** | All-cause hospitalisation (3 [5.1%])  Cardiovascular hospitalisation (2 [3.4%])  Heart failure hospitalisation (2 [3.4%])  Permanent institutionalisation (1 [1.7%]) |
| **Impact (3 [5.1%])** | CIRS (1 [1.7%])  MCSI (1 [1.7%])  MSPSS (1 [1.7%]) |
| **Disability (2 [3.4%])** | Barthel index (2 [3.4%])  Falls efficacy scale (1 [1.7%]) |
| **Sleep (2 [3.4%])** | ESS (1 [1.7%])  Polysomnography (1 [1.7%])  SASQ (1 [1.7%]) |
| **Specific (2 [3.4%])** | ACQ (1 [1.7%])  PAID (1 [1.7%])  SDSCA (1 [1.7%]) |
| **Economic (1 [1.7%])** | ICER (1 [1.7%]) |

**Note: studies in the ‘both’ categories are also accounted for in the field-/lab-based categories for exercise capacity, and general/disease-specific categories for health-related quality of life outcome domains.*

# **Table S2.** Outcome measure abbreviations.

| **Abbreviation** | **Definition** |
| --- | --- |
| 15D | 15-Dimensional health-related quality of life instrument |
| 12MWT | Twelve-Minute Walk Test |
| 1RM | One-repetition maximum |
| 25(OH)D | 25-hydroxy-vitamin D |
| 2MST | Two-Minute Step Test |
| 2MWT | Two-Minute walk test |
| 3MST | Three-Minute Step Test |
| 6mWS | Six-meter Walk Speed |
| 6MWT | Six-Minute walk test |
| Aβ42 | Amyloid beta 42 |
| AAP | Adelaide Activities Profile |
| ABCS | Activities-Specific Balance Confidence Scale |
| ACIF | Acute Care Index of Function |
| ACQ | Asthma Control Questionnaire |
| ACTH | Adrenocorticotropic hormone |
| ADCS ADL | Alzheimer’s Disease Cooperative Study – Activities of Daily Living scale |
| ADL | Activities of Daily Living |
| ADMA | Asymmetrical Dimethylarginine |
| ADQRL | Alzheimer’s Disease Quality Related Life Scale |
| AF | Atrial Fibrilation |
| AFEQT | Atrial Fibrilation Effect on Quality of life |
| AFI | Arthritis Function Index |
| AF-QOL-18 | Atrial Fibrillation Quality of Life 18 |
| AIMS | Arthritis Impact Measurement Scales |
| ALP | Alkaline Phosphatase |
| ALT | Alanine aminotransferase |
| AMH | Anti-Mullerian Hormone |
| APQLQ | Angina Pectoris Quality of Life Questionnaire |
| AQLQ | Asthma Quality of Life Questionnaire |
| AQOL | Assessment of Quality of Life instrument |
| ARAT | Action Research Arm Test |
| ARSW | Adjective Rating Scale for Withdrawal |
| ASI | Addiction Severity Index |
| AST | Aspartate aminotransferase |
| AT | Anaerobic threshold |
| ATAQ-IPF | A Tool to Assess QoL in IPF |
| ATQ | Automatic Thoughts Questionnaire |
| BADS | Behavioural Activation for Depression Scale |
| BAI | Beck Anxiety Inventory |
| BDI | Baseline Dyspnoea Index / Beck Depression Inventory |
| BDNF | Brain Derived Neurotrophic Factor |
| BESTest | Balance Evaluation Systems Test |
| BFI | Brief Fatigue Inventory |
| BMD | Bone Mineral Density |
| BMI | Body Mass Index |
| BNP | B-type natriuretic peptide |
| BPI | Brief Pain Inventory |
| BRS | Baroreflex Sensitivity |
| BSPS | Brief Social Phobia Scale |
| CABG | Coronary Artery Bypass Graft |
| CAT | Chronic Obstructive Pulmonary Disease (COPD) Assessment Test |
| CDAD(-IADL) | Chinese Disability Assessment for Dementia (Instrumental Activities of Daily Living) |
| CDAI | Crohn's Disease Activity Index |
| CDT | Carbohydrate-Deficient Transferrin |
| CES-D | Centre for Epidemiological Studies Depression Scale |
| CFQ-R | Cystic Fibrosis Questionnaire-Revised |
| CGI | Clinical Global Impressions |
| CHAMPS | Community Healthy Activities Model Programme for Seniors |
| CIRS | Chronic Illness Resource Survey |
| CIS/CIS20r | Checklist Individual Strength |
| CMSA | Chedoke-McMaster Stroke Assessment |
| COWS | Clinical Opiate Withdrawal Scale |
| CPCI | Chronic Pain Coping Inventory |
| CPET | Cardiopulmonary Exercise Test |
| CPGS | Chronic Pain Grade Scale |
| CPSS | Chronic Pain Self-Efficacy Scale |
| CRF | Cancer Related Fatigue |
| CRP | C-Reactive Protein |
| CRQ | Chronic Respiratory Disease Questionnaire |
| CSA | Cross-Sectional Area |
| CS-PFP10 | 10-item Continuous Scale Physical Functional Performance Test |
| CV | Cardiovascular |
| CWR | Constant Work Rate |
| DAS-28 | Disease Activity Score for rheumatoid arthritis |
| DASI | Duke Activity Status Index |
| DAST | Drug Abuse Screening Test |
| DBP | Diastolic Blood Pressure |
| DDS17 | Diabetes Distress Scale 17 |
| DEXA | Dual Energy X-ray Absorptiometry |
| DHEA-S | Dehydroepiandrosterone Sulfate |
| D-QOL | Dementia Quality of Life questionnaire |
| EE | Energy Expenditure |
| eGFR | Estimated Glomerular Filtration Rate |
| EORTC QLQ-C30 | European Organisation for Research  and Treatment of Cancer Quality of  Life Questionnaire Core 30 |
| EORTC QLQ-PR | Prostate specific module of European Organisation for Research  and Treatment of Cancer Quality of  Life Questionnaire |
| EPIC | Expanded Prostate Cancer Index Composite |
| EPQ | Eysenck Personality Questionnaire |
| EQ-5D | EuroQol 5 Dimensions |
| ERV | Expiratory Reserve Volume |
| ESAS | Edmonton Symptom Assessment Scale |
| ESS | Epworth Sleepiness Scale |
| ESWT | Endurance Shuttle Walk Test |
| FAB | Fullerton Advance Balance scale |
| FAC | Functional Ambulation Classification |
| FACT (-P, -G, -F) | Functional Assessment of Cancer  Therapy (P, prostate; G, general; F, fatigue) |
| FAI | Free Androgen Index |
| FAPS | Functional Ambulation Profile Score |
| FAS | Fibromyalgia Activity Score |
| FAST | Fitness Arthritis and Seniors Trial |
| FCP | Fecal Calprotectin |
| FE_NO_ | Exhaled Fraction of Nitric Oxide |
| FEV_1_ (%) | Forced Expiratory Volume in 1 second (% predicted) |
| FFABQ | Fear of Falling Avoidance Questionnaire |
| FFMQ-SF | Five Facet Mindfulness Questionnaire-Short Form |
| FICSIT-4 | Frailty and Injuries Cooperative Studies of Intervention Techniques -subtest 4 |
| FIM | Functional Independence Measure |
| FIQ | Fibromyalgia Impact Questionnaire |
| FIS | Fatigue Impact Scale |
| FMD | Flow-Mediated Dilation |
| fMRI | Functional Magnetic Resonance Imaging |
| FOSQ | Functional Outcomes of Sleep Questionnaire |
| FPI | Functional Performance Inventory |
| FQ | Fatigue Questionnaire |
| FROP-Com | Falls Risk for Older People - Community |
| FSMC | Fatigue Scale for Motor and Cognitive Functions |
| FSQ | Functional Status Questionnaire |
| FSS | Fatigue Severity Scale |
| FTHUE | Functional Test of the Hemiparetic Upper Extremity |
| FVC (%) | Forced Vital Capacity (% predicted) |
| GAD-7 | General Anxiety Disorder 7-item Scale |
| GARS | Groningen Activity Restriction Scale |
| GCHFQ | Guyatt Chronic Heart Failure Questionnaire |
| GDS | Geriatric Depression Scale |
| GHQ | General Health Questionnaire |
| GT | Gammaglutamyltransferase |
| HADS | Hospital Anxiety and Depression Scale |
| HAM-A | Hamilton Anxiety Rating Scale |
| HAM-D | Hamilton Depression Rating Scale |
| HAQ | Health Assessment Questionnaire |
| HbA1c | Haemoglobin A1c |
| HBI | Harvey-Bradshaw Index |
| HDL | High-Density Lipoprotein |
| HF | Heart Failure |
| HGS | Hand-grip Strength |
| HLQ | Health and Labour Questionnaire |
| HOMA-IR | Homeostatic Model Assessment for Insulin Resistance |
| HQoL | Hacettepe Quality of Life |
| HR | Heart Rate |
| HRR | Heart Rate Recovery |
| HRV | Heart Rate Variability |
| HSCL-25 | Hopkins Symptom Checklist-25 |
| hsCRP | High Sensitivity C-reactive Protein |
| IBDQ | Inflammatory Bowel Disease Questionnaire |
| ICER | Incremental Cost-Effectiveness Ratio |
| ICQ | Intermittent Claudication Questionnaire |
| IGF-1 | Insulin-like Growth Factor 1 |
| IL | Interleukin |
| IPAQ | International Physical Activity Questionnaire |
| IRGL | Influence of Rheumatic Diseases on General Health and Lifestyle questionnaire |
| ISWT | Incremental Shuttle Walk Test |
| JOA | Japanese Orthopedic Association |
| K6 | Kessler Psychological Distress scale 6-item |
| KBILD | King’s Brief Interstitial Lung Disease questionnaire |
| KCCQ | Kansas City Cardiomyopathy Questionnaire |
| KDQOL-SF | Kidney Disease Quality of Life Short Form |
| KOOS | Knee Osteoarthritis Outcome Scale |
| LAVI | Left Atrial Volume Index |
| LBP | Low Back Pain |
| LCQ | Leicester Cough Questionnaire |
| LDL | Low-Density Lipoprotein |
| LLFDI | Late Life Function and Disability Instrument |
| LOT-R | Life Orientation Test-Revised |
| LSI | Life Satisfaction Index |
| LVEF | Left Ventricular Ejection Fraction |
| LVMI | Left Ventricular Mass Index |
| MAP | Mean Arterial Pressure |
| MCSI | Modified Caregiver Strain Index |
| MCTSIB | Modified Clinical Test of Sensory Interaction of Balance |
| MEP | Maximal Expiratory Pressure |
| METs | Metabolic Equivalent of Tasks |
| MFI | Multidimensional Fatigue Inventory |
| MHC | Myosin Heavy-Chain |
| MIP | Maximal Inspiratory Pressure |
| MLHFQ | Minnesota Living with Heart Failure  questionnaire |
| MMPI | Minnesota Multiphasic Personality Inventory |
| (m)MRC | (modified) Medical Research Council Dyspnoea Scale |
| MoCA | Montreal Cognitive Assessment |
| MOLBPQ | Modified Oswestry Disability Index Low Back Pain Questionnaire |
| MOS | Medical Outcomes Study |
| MPPB | Modified Physical Performance Battery |
| MSPSS | Multidimensional Scale for Perceived Social Support |
| MSWS-12 | Multiple Sclerosis Walking Scale |
| MVC | Maximum Voluntary Contraction |
| MVPA | Moderate-to-Vigorous Physical Activity |
| MVV | Maximum Voluntary Ventilation |
| NEADL | Nottingham Extended Activities of Daily Living |
| NHP | Nottingham Health Profile |
| NIRS | Near-Infrared Spectroscopy |
| NOSGER | Nurses Observation Scale for Geriatric Patients |
| NRS | Numerical Rating Scale |
| NT-pro-BNP | N-terminal pro-B-type natriuretic peptide |
| OARS | Older Americans Resources and Services questionnaire |
| OASI | Osteoarthritis Screening Index |
| ODI | Oswestry Disability Index |
| OMPSQ | Orebro Musculoskeletal Pain Screening Questionnaire |
| OPAQ | Osteoporosis Assessment Questionnaire |
| OUES | Oxygen Uptake Efficiency Slope |
| PA | Physical Activity |
| PACER | Progressive Aerobic Cardiovascular Endurance Run |
| PaCO_2_ | Partial arterial carbon dioxide pressure |
| PADS | Physical Activity and Disability Scale |
| PAID | Problem Areas in Diabetes scale |
| PANAS | Positive and Negative Affect Schedule |
| PANSS | Positive and Negative Syndrome Scale |
| PaO_2_ | Partial arterial oxygen pressure |
| PASE | Physical Activity Scale for the Elderly |
| PASIPD | Physical Activity Scale for Individuals with Physical Disabilities |
| PASS | Physical Activity Status Scale |
| PCI | Percutaneous Coronary Intervention |
| PCOS-Q | Polycystic Ovary Syndrome Quality of Life scale |
| PDQ-39 | Parkinson's Disease Questionnaire |
| PEF | Peak Expiratory Flow |
| PF4 | Platelet Factor 4 |
| PFS | Piper Fatigue Scale |
| PGAQoL | Patient's Global Assessment of Quality of Life |
| PHQ-9 | Patient Health Questionnaire |
| PINP | Pro-collagen type-I N-terminal Propeptide |
| POMA | Performance Oriented Mobility Assessment |
| POMS | Profile of Mood States |
| PPA | Physiological Profile Assessment |
| PROMIS-29 | Patient-Reported Outcomes Measurement Information System-29 |
| PSA | Prostate Specific Antigen |
| PSFS | Patient Specific Functional Scale |
| PSQI | Pittsburgh Sleep Quality Index |
| PSS | Perceived Stress Scale |
| QALY | Quality-Adjusted Life Year |
| QBPDS | Quebec Back Pain Disability Scale |
| QIDS | Quick Inventory of Depression Symptomology |
| QLI | Quality of Life Index |
| QLMI | Quality of Life after Myocardial Infarction |
| QOL DA | Quality of Life Drug Addiction dependence |
| QUALEFFO-41 | 41-Item Quality of Life Questionnaire of the European Foundation for Osteoporosis |
| QUALID | Quality of Life in Late-stage Dementia |
| REPDS | Revised Elderly Persons Disability Scale |
| RER | Respiratory Exchange Ratio |
| RLOC | Recovery Locus of Control Scale |
| RMA | Rivermead Motor Assessment |
| RMI | Rivermead Mobility Index |
| ROM | Range of Motion |
| RPE | Rating of Perceived Exhaustion |
| RSES | Rosenberg Self-Esteem Scale |
| SADD | Short Alcohol Dependence Data questionnaire |
| SaO_2_ | Oxygen saturation of arterial blood |
| SarQol | Sarcopenia and Quality of Life Questionnaire |
| SAS | Self-rating Anxiety Scale |
| SASQ | Sleep Apnoea Syndrome Questionnaire |
| SBP | Systolic Blood Pressure |
| SCCAI | Simple Clinical Colitis Activity Index |
| SCL-90-R | Symptom Checklist-90-Revised |
| SCQ | Situational Confidence Questionnaire |
| SDF-1 | Stromal cell Derived Factor-1 |
| SDS | Self-rating Depression Scale |
| SDSCA | Summary of Diabetes Self-Care Activities |
| SE-ADL | Schwab and England Activities of Daily Living scale |
| SEES | Subjective Exercise Experience Scale |
| SF-8 | 8-Item Short Form Survey |
| SF-12 | 12-Item Short Form Survey |
| SF-36 | 36-Item Short Form Survey |
| SGRQ | St. George's Respiratory Questionnaire |
| SHBG | Sex Hormone Binding Globulin |
| SIGHD | Structured Interview Guide for Hamilton Depression scale |
| SIP | Sickness Impact Profile |
| SIS | Stroke Impact Scale |
| SMAST | Short Michigan Alcohol Screening Test |
| SPPB | Short Physical Performance Battery |
| SS-QOL | Stroke Specific Quality of Life |
| SSS | Scandinavian Stroke Scale |
| STAI | State-Trait Anxiety Index |
| SV | Stroke Volume |
| SVS | Subjective Vitality Scale |
| SWLS | Satisfaction with Life Scale |
| T10MW | Timed 10m Walk |
| T25FW | Timed 25ft Walk |
| T50MW | Timed 50m Walk |
| TFLB | Timeline Followback Method |
| TGF-b | Transforming Growth Factor-beta |
| TLC | Total Lung Capacity |
| TLCO | Transfer factor for carbon monoxide |
| TNF-a | Tumour Necrosis Factor-alpha |
| TUG | Timed Up and Go |
| UCSD SOBQ | University of California San Diego Shortness of Breath Questionnaire |
| UPDRS | Unified Parkinson's Disease Rating Scale |
| VAS | Visual Analogue Scale |
| V_E_ | Minute Ventilation |
| VCO_2_ | Volume of carbon dioxide production |
| VEGF | Vascular Endothelial Growth Factor |
| VO_2_ | Volume of Oxygen consumption |
| WELCH | Walking Estimated-Limitation Calculated by History |
| WHOQOL | World Health Organisation Quality of Life assessment |
| WHYMPI | West Haven-Yale Multidimensional Pain Inventory |
| WIQ | Walking Impairment Questionnaire |
| WOMAC | Western Ontario and McMaster Universities Osteoarthritis Index |
| WR | Work Rate |
| YPAS | Yale Physical Activity Survey |
| YKL-40 | Chitinase 3-like-protein-1 |

# **Table S3.** Outcome measures reported across exercise-based rehabilitation research in MLTC organised into core outcome set for multimorbidity (COSmm) domains.

| **Studies** | **COSmm domains** | | | | | | | | | | | |
| --- | --- | --- | --- | --- | --- | --- | --- | --- | --- | --- | --- | --- |
|  | **HRQoL / Self-rated health** | **Mental health** | **Mortality** | **Treatment burden** | **Self- management behaviour** | **Self-efficacy** | **Adherence** | **Activities of daily living** | **Physical function** | **Physical activity** | **Health care use** | **Costs** |
| Abd El- Kader et al. (2013) |  |  |  |  |  |  |  |  |  |  |  |  |
| Abdelbasset et al. (2019) |  | PHQ-9 |  |  |  |  |  |  |  |  |  |  |
| Al-Jiffri et al. (2013) |  |  |  |  |  |  |  |  |  |  |  |  |
| Asa et al. (2012) | SF-36 MLHFQ | HADS HAM-D |  |  |  |  | Sessions attended |  | WRpeak VO2peak 6MWT n heel lifts n shoulder flexion n shoulder abduction HGS Peak torque knee Isometric strength |  |  |  |
| Barnes et al. (2009) | SF-36 FOSQ | BDI POMS |  |  |  |  |  |  | VO2peak Wrmax Hrisowork |  |  |  |
| Beaudoin et al. (2017) | CFQ-R |  |  |  |  |  |  |  | VO2peak VE at VO2peak N push up n sit up Leg press 1RM Chest press 1RM Lat pulldown 1RM Bicep curl 1RM HGS | Daily step count Total EE Unsp. PA questionnaire |  |  |
| Bernocchi et al. (2018) | MLHFQ CAT |  |  |  |  |  | Adherence | Barthel  index | 6MWT | PASE |  |  |
| Blumenthal et al. (2012) |  | BDI-II | All-cause mortality CV mortality HF mortality |  |  |  | Adherence |  | VO2peak |  | All-cause hospitalisation CV hospitalisation HF hospitalisation |  |
| Blumenthal et al. (2012) |  | HAM-D |  |  |  |  | Sessions  attended |  | VO2peak Treadmill duration |  |  |  |
| Byrkjeland et al. (2015) |  |  |  |  |  |  | Adherence |  | VO2peak VT CPET  Time to exhaustion |  |  |  |
| Campo et al. (2020) | EQ-5D |  |  |  |  |  |  |  | 10m gait speed HGS |  |  |  |
| Castro et al. (2015) |  |  |  |  |  |  |  |  | 6MWT |  |  |  |
| Chiang et al. (2020) | SF-36 |  |  |  |  |  |  |  | VO2peak | IPAQ |  |  |
| Collins et al. (2010) |  | Depressive  symptoms |  |  |  |  |  |  |  |  |  |  |
| Crisafulli et al. (2010) | SGRQ |  |  |  |  |  | Programme  completers |  | 6MWT |  |  |  |
| de Groot et al. (2012) | SF-36 Diabetes QoL | BDI |  |  | CIRS |  |  |  | VO2peak | Min exercise/week Steps/week Godin activity |  |  |
| de Groot et al. (2019) | SF-12 Diabetes QoL | BDI-II Automatic thoughts questionnaire Diabetes distress |  |  |  |  |  |  | 6MWT |  |  |  |
| Edelmann et al. (2011) / Nolte (2015) | SF-36 MLHFQ | PHQ-9 |  |  |  |  |  |  | VO2peak 6MWT Wrmax AT VO2 AT WR |  |  |  |
| Freitas et al. (2018) | AQLQ |  |  |  |  |  |  |  | VO2peak Wrmax Quad strength | Steps/day |  |  |
| Gary et al. (2004) | MLHFQ | GDS |  |  |  |  | Adherence |  | 6MWT DASI |  |  |  |
| Gary et al. (2010) | MLHFQ | HAM-D |  |  |  |  | Adherence |  | 6MWT |  |  |  |
| Gary et al. (2012) | KCCQ | BDI |  |  |  |  | Adherence |  | 6MWT HGS Forearm flexion Knee extension CS-PFP10 |  |  |  |
| Gretebeck et al. 2019 |  |  |  |  |  |  | Attendance |  | 6MWT Comfortable gait speed | CHAMPS |  |  |
| Halvari et al. (2017) | SF-36 SVS |  |  |  |  |  | Attendance |  |  |  |  |  |
| Hassan et al. (2016) | SGRQ |  |  |  |  |  |  |  | 6MWT Estimated VO2max |  |  |  |
| Hinrichs et al. (2016) | SF-8 |  |  |  |  | Exercise  self-efficacy Falls efficacy scale | Adherence |  | 2-minute step test Sit-to-stand HGS TUG Sit-and-reach Tandem stand Tandem walk | Steps/day |  |  |
| Hsu et al. (2011) - now Hsu et al. (2021) | WOMAC |  |  |  |  |  | Adherence |  | TUG |  |  |  |
| Johnson et al. (2014) |  |  |  |  |  |  |  |  | 6MWT Sit-to-stand |  |  |  |
| Keihani et al. (2014) | SF-36 | BDI BAI |  |  |  |  |  |  |  |  |  |  |
| Khadanga et al. (2016) | SF-36 | GDS |  |  |  |  |  |  | VO2peak METSpeak |  |  |  |
| Koukouvou et al. (2004) | QLI MLHFQ Scale of Life Satisfaction | BDI HADS EPQ |  |  |  |  | Sessions  attended |  | VO2peak VE/VO2peak VO2 AT RER Hrmax/peak Vemax Cycle time |  |  |  |
| Kulcu et al. (2007) | HQoL | BDI STAI |  |  |  |  |  |  | Modified Bruce protocol MET Hrmax VO2 AT VO2peak |  |  |  |
| Kurian et al. (2010) |  |  |  |  |  |  |  |  |  |  |  |  |
| Leung et al. (2013) | CRQ | HADS |  |  |  |  | Sessions  attended |  | ISWT ESWT Isokinetic quad strength Body sway Functional reach MPPB FPI |  |  |  |
| Leung et al. (2019) | SF-12 | PSS-10 |  |  |  | Tai-Chi  exercise self-efficacy | Attendance |  |  |  |  |  |
| Listerman et al. (2011) | SF-36 |  |  |  |  |  |  |  | 6MWT |  |  |  |
| Lo et al. (2021) |  |  |  |  |  |  |  |  | VO2max AT WR HGS Sit-to-stand Sit-and-reach | IPAQ |  |  |
| Martin et al. (2016) |  |  | Mortality |  |  |  | Attendance Completion |  | METS |  |  |  |
| McNamara et al. (2013) | CRQ | HADS |  |  |  |  | Attendance |  | 6MWT ISWT ESWT MIP MIP%pred MEP MEP%pred |  |  |  |
| Mentz et al. (2013) | KCCQ |  | All-cause mortality CV mortality |  |  |  | Adherence |  | 6MWT CPET time VO2peak |  | All-cause hospitalisation CV hospitalisation HF hospitalisation |  |
| Mesquita et al. (2015) | SGRQ |  |  |  |  |  |  |  | 6MWT CWR cycling time |  |  |  |
| Mundra et al. (2013) |  | BDI |  |  |  |  |  |  | METS |  |  |  |
| Naz et al. (2019) | SGRQ SF-36 | HADS |  |  |  |  |  |  | 6MWT |  |  |  |
| Nonoyama et al. (2016) |  |  |  |  |  |  |  |  | VO2peak Wrmax Ventilatory anaerobic threshold |  |  |  |
| Oerkid et al. (2012) | SF-12 | HADS |  |  |  |  |  |  | 6MWT Sit-to-stand |  |  |  |
| Pibernik-Okanović et al. (2015) | SF-12 | PAID CES-D |  |  | SDSCA |  |  |  |  |  |  |  |
| Rodriguez-Mañas et al. (2019) | EQ-5D-5L |  | Mortality | Modified Caregiver Strain Index |  |  | Adherence | Barthel  index | SPPB |  | Hospitalisation Permanent institutionalisation | ICER |
| Schneider et al. (2016) |  | HAM-D BDI-II BADS |  | Multidimensional Scale for Perceived Social Support |  | Self-efficacy  for exercise scale | Adherence |  |  | Accelerometer MVPA |  |  |
| Servantes et al. (2012) | MLHFQ |  |  |  |  |  | Adherence |  | VO2peak VE/VCO2 VO2 AT Isokinetic quad strength |  |  |  |
| Soleimani et al. (2009) |  |  |  |  |  |  |  |  |  |  |  |  |
| Soliman and Abdelbasset (2019) |  | PHQ-9 |  |  |  |  |  |  |  |  |  |  |
| Sridhar (2010) |  |  |  |  |  |  |  |  |  |  |  |  |
| Srinivasan (2014) |  | SIGHD |  |  |  |  |  |  |  |  |  |  |
| Takaya (2014) |  |  |  |  |  |  | Attendance |  | Peak RER VO2peak VE/VCO2 HRR |  |  |  |
| Tunsupon (2017) | CRQ |  |  |  |  |  |  |  | 6MWT CWR cycling time |  |  |  |
| Verges (2004) |  |  |  |  |  |  | Attendence |  | WRpeak Exercise time Hrmax VO2peak AT |  |  |  |
| Wang (2013) |  |  |  |  |  |  |  |  | VO2peak VO2 VT Wrmax WR VT Vepeak VE VT VCO2peak VCO2 VT VE/VCO2 slope OUES Hrpeak Svpeak |  |  |  |
| Woodard (1994) |  |  |  |  |  |  | Attendance |  | METS |  |  |  |
| Zwerink (2010) | MLHFQ CRQ |  |  |  |  |  |  |  | 6MWT ISWT |  |  |  |
| **Total number of studies** | 35 | 26 | 4 | 2 | 2 | 3 | 25 | 2 | 45 | 9 | 3 | 1 |


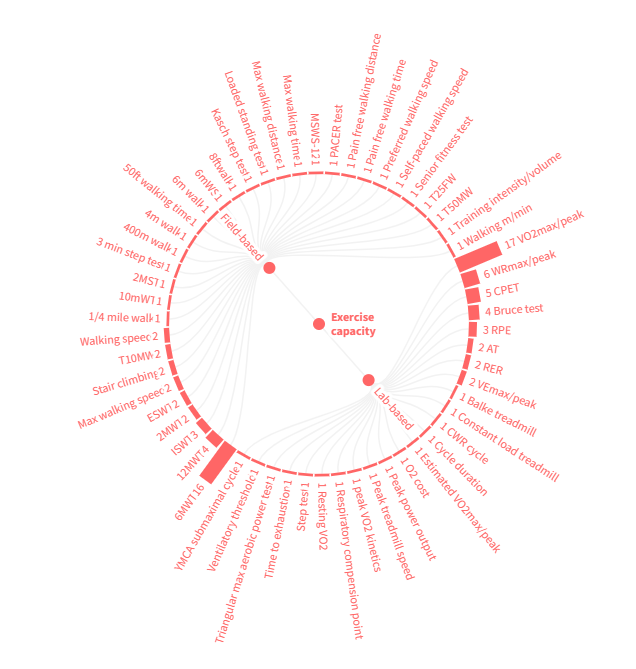


**a)**


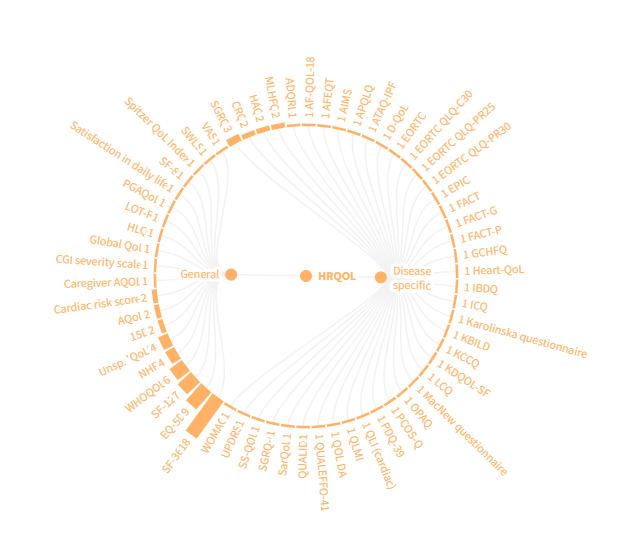


**b)**

# **Figure S1.** Outcome measures reported within exercise-rehabilitation research across single long-term conditions (LTC) organised into (a) exercise capacity (across 22 LTC) and (b) health-related quality of life (across 22 LTC) domains.

***Note:*** *numbers represent total number of LTC reporting a particular outcome measure. Abbreviations are outlined in table S2.*

# **Table S4.** Outcome domains and measures reported across exercise-based rehabilitation systematic reviews for 25 single long-term conditions (LTC).

| **Outcome domains (number [%] LTC)** | **Outcome domains (number [%] LTCs)** |
| --- | --- |
| **Exercise capacity (22 [88%])** | **Field-based:**  6MWT (16 [64%])  12MWT (4 [16%])  ISWT (3 [12%])  2MWT (2 [8%])  ESWT (2 [8%])  Max walking speed (2 [8%])  Stair climbing (2 [8%])  T10MW (2 [8%])  Walking speed (2 [8%])  ¼ mile walk (1 [4%])  10Mwt (1 [4%])  2MST (1 [4%])  3MST(1 [4%])  400m walk (1 [4%])  4m walk (1 [4%])  50ft walking time (1 [4%])  6m walk (1 [4%])  6mWS (1 [4%])  8ft walk (1 [4%])  Kasch step test (1 [4%])  Loaded standing test (1 [4%])  Max walking distance (1 [4%])  Max walking time (1 [4%])  MSWS-12 (1 [4%])  PACER test (1 [4%])  Pain free walking distance (1 [4%])  Pain free walking time (1 [4%])  Preferred walking speed (1 [4%])  Self-paced walking speed (1 [4%])  Senior fitness test (1 [4%])  T25FW (1 [4%])  T50MW (1 [4%])  Training intensity/volume (1 [4%])  Walking m/min (1 [4%])  **Lab-based:**  VO_2_max/peak (17 [68%])  WRmax/peak (6 [24%])  CPET (5 [20%])  Bruce test (3 [12%])  RPE (3 [12%])  AT (2 [8%])  RER (2 [8%])  V_E_max/peak (2 [8%])  Balke treadmill (1 [4%])  Constant load treadmill (1 [4%])  CWR cycle (1 [4%])  Cycle duration (1 [4%])  Estimated VO_2_max/peak (1 [4%])  O_2_ cost (1 [4%])  Peak power output (1 [4%])  Peak treadmill speed (1 [4%])  Peak VO_2_ kinetics (1 [4%])  Respiratory compensation point (1 [4%])  Resting VO_2_ (1 [4%])  Step test (1 [4%])  Time to exhaustion (1 [4%])  Triangular max aerobic power test (1 [4%])  Ventilatory threshold (1 [4%])  YMCA submaximal cycle (1 [4%]) |
| **Health-related quality of life (22 [88%])** | **General:**  SF-36 (18 [72%])  EQ-5D (9 [36%])  SF-12 (7 [28%])  WHOQOL (6 [24%])  NHP (4 [16%])  Unspecified ‘QoL’ (4 [16%])  15D (2 [8%])  AQoL (2 [8%])  Cardiac risk score (2 [8%])  Caregiver QoL (1 [4%])  CGI severity (1 [4%])  Global QoL (1 [4%])  HLQ (1 [4%])  LOT-R (1 [4%])  PGAQoL (1 [4%])  Satisfaction in daily life (1 [4%])  SF-8 (1 [4%])  Spitzer QoL index (1 [4%])  SWLS (1 [4%])  VAS (1 [4%])  **Disease specific:**  SGRQ (3 [12%])  CRQ (2 [8%])  HAQ (2 [8%])  MLHFQ (2 [8%])  FACT (1 [4%])  WOMAC (1 [4%])  ADQRL (1 [4%])  AF-QoL-18 (1 [4%])  AFEQT (1 [4%])  AIMS (1 [4%])  APQLQ (1 [4%])  ATAQ-IPF (1 [4%])  D-QoL (1 [4%])  EORTC (1 [4%])  EORTC QLQ-C30 (1 [4%])  EORTC QLQ-PR25 (1 [4%])  EORTC QLQ-PR 30 (1 [4%])  EPIC (1 [4%])  FACT-G (1 [4%])  FACT-P (1 [4%])  GCHFQ (1 [4%])  Heart QoL (1 [4%])  IBDQ (1 [4%])  ICQ (1 [4%])  Karolinska questionnaire (1 [4%])  KBILD (1 [4%])  KCCQ (1 [4%])  KDQOL-SF (1 [4%])  LCQ (1 [4%])  MacNew questionnaire (1 [4%])  OPAQ (1 [4%])  PCOS-Q (1 [4%])  PDQ-39 (1 [4%])  QLI cardiac (1 [4%])  QLMI (1 [4%])  QoL DA (1 [4%])  QUALEFFO-41 (1 [4%])  QUALID (1 [4%])  SarQoL (1 [4%])  SGRQ-I (1 [4%])  SS-QOL (1 [4%])  UPDRS (1 [4%]) |
| **Strength (17 [68%])** | **Lower limb:**  Sit-to-stand (13 [52%])  Lower-limb 1RM (5 [20%])  Knee extension (4 [16%])  Knee flexion (3 [12%])  Leg press (3 [12%])  Ankle plantarflexion (2 [8%])  Dynamic strength (2 [8%])  Isometric strength (2 [8%])  Leg strength (2 [8%])  Ankle dorsiflexion (1 [4%])  Ankle flexion (1 [4%])  Ankle peak torque (1 [4%])  Bilateral lower limb extension force (1 [4%])  Composite strength score (1 [4%])  Hip extension/flexion (1 [4%])  Isokinetic fatigue score (1 [4%])  Isokinetic peak torque (1 [4%])  Leg 10RM (1 [4%])  Leg press number of repetitions (1 [4%])  Lower limb strength (1 [4%])  Maximum isometric force (1 [4%])  Maximum quadriceps strength (1 [4%])  Maximum unilateral force (1 [4%])  Maximum weight bearing (1 [4%])  MVC (1 [4%])  Paretic knee strength (1 [4%])  Quadriceps force (1 [4%])  Rate of force development (1 [4%])  Static squat (1 [4%])  Vertical jump (1 [4%])  **Upper body:**  HGS (11 [44%])  Upper body 1RM (3 [12%])  Arm curl (2 [8%])  Chest press (2 [8%])  Elbow flexion (2 [8%])  Abdominal strength test (1 [4%])  Back extension (1 [4%])  Bench press (1 [4%])  Dynamic strength (1 [4%])  Dynamometer (1 [4%])  Elbow extension (1 [4%])  Push up test (1 [4%])  Seated medicine ball throw (1 [4%])  Sit-ups time (1 [4%])  Static arm pull (1 [4%])  Trunk strength (1 [4%])  **Respiratory:**  MIP (2 [8%])  MEP (2 [8%])  Manual muscle test diaphragm (1 [4%])  **Unspecified:**  ‘Muscle strength’ (2 [8%])  MVC (1 [4%]) |
| **Biomarkers (15 [60%])** | Glucose (4 [16%])  HDL (4 [16%])  Total cholesterol (4 [16%])  Triglycerides (4 [16%])  Lactate (3 [12%])  LDL (3 [12%])  Cholesterol (2 [8%])  Cortisol (2 [8%])  CRP (2 [8%])  Haematocrit (2 [8%])  Haemoglobin (2 [8%])  HbA1c (2 [8%])  HOMA-IR (2 [8%])  hsCRP (2 [8%])  Insulin (2 [8%])  Leukocyte count (2 [8%])  Prolactin (2 [8%])  ACTH (1 [4%])  Adiponectin (1 [4%])  ADMA (1 [4%])  Albumin (1 [4%])  AMH (1 [4%])  Aβ42 (1 [4%])  BDNF (1 [4%])  Brain atrophy (1 [4%])  Carnitine (1 [4%])  CDT (1 [4%])  Creatine (1 [4%])  Creatinine (1 [4%])  DHEA-S (1 [4%])  Dopamine (1 [4%])  Endogenous NO products (1 [4%])  Endothelial progenitor cells (1 [4%])  Erythrocyte elongation index (1 [4%])  Erythrocyte sedimentation rate (1 [4%])  F2-isoprostanes (1 [4%])  FAI (1 [4%])  FCP (1 [4%])  Fibrinogen (1 [4%])  fMRI (1 [4%])  Follicle stimulating hormone (1 [4%])  Glucose control (1 [4%])  Glycaemia (1 [4%])  GT (1 [4%])  IGF-1 (1 [4%])  IL-6 (1 [4%])  Immune function (1 [4%])  Insulin sensitivity (1 [4%])  Lipid profile (1 [4%])  Luteinising hormone (1 [4%])  MHC isoforms (1 [4%])  Microalbuminuria (1 [4%])  Neutrophil adhesion (1 [4%])  Neutrophil count (1 [4%])  NIRS (1 [4%])  NT-proBNP (1 [4%])  Oestradiol (1 [4%])  Phosphorus (1 [4%])  Plasma lipoproteins (1 [4%])  Plasminogen activator inhibitor (1 [4%])  Potassium (1 [4%])  Progesterone (1 [4%])  Prothrombin fragments (1 [4%])  PSA (1 [4%])  Salivary alpha-amylase (1 [4%])  SDF-1 (1 [4%])  SHBG (1 [4%])  Testosterone (1 [4%])  Thrombin antithrombin complex (1 [4%])  Tissue plasminogen activator (1 [4%])  TNF-a (1 [4%])  Unspecified ‘inflammatory biomarkers’ (1 [4%])  Urea (1 [4%])  Urine morphine (1 [4%])  VEGF (1 [4%]) |
| **Depression (15 [60%])** | HADS (12 [48%])  BDI (6 [24%])  CES-D (4 [16%])  SDS (3 [12%])  GDS (2 [8%])  PHQ-9 (2 [8%])  AIMS (1 [4%])  HAM-D (1 [4%])  HAM-D17 (1 [4%])  QIDS (1 [4%])  VAS (1 [4%]) |
| **Body composition (15 [60%])** | BMI (9 [36%])  Body fat % (5 [20%])  Body weight (4 [16%])  Waist circumference (3 [12%])  Waist-hip ratio (3 [12%])  Body fat (2 [8%])  Lean mass (2 [8%])  Muscle mass (2 [8%])  Muscle mass % (2 [8%])  Weight (2 [8%])  BMD (1 [4%])  Body mass (1 [4%])  DEXA body composition (1 [4%])  Fat free mass (1 [4%])  Fat mass (1 [4%])  Ferriman-Gallwey (1 [4%])  Fibre area (1 [4%])  Fibre type distribution (1 [4%])  Height (1 [4%])  Hip circumference (1 [4%])  Lumbar lordosis (1 [4%])  Mid-thigh CSA (1 [4%])  Muscle capillarisation (1 [4%])  Muscle CSA (1 [4%])  Muscle volume (1 [4%])  Thigh circumference (1 [4%])  Thoracic kyphosis (1 [4%])  Triceps skinfold (1 [4%]) |
| **Anxiety (14 [56%])** | HADS (10 [40%])  STAI (5 [20%])  BAI (4 [16%])  GAD-7 (2 [8%])  PSS (2 [8%])  SAS (2 [8%])  BSPS (1 [4%])  HAM-A (1 [4%])  IBD stress index (1 [4%]) |
| **Specific (13 [52%])** | Alcohol TFLB (2 [8%])  ADCS ADL (1 [4%])  AF symptoms/severity questionnaire (1 [4%])  Agonist activity (1 [4%])  Alcohol consumption (1 [4%])  Alcohol urge questionnaire (1 [4%])  Ankle-brachial index (1 [4%])  Antagonist activity (1 [4%])  ARSW (1 [4%])  Arthritis self-efficacy score (1 [4%])  ASI (1 [4%])  Brief self-control scale (1 [4%])  CABG revascularisation (1 [4%])  CDAI (1 [4%])  CMSA (1 [4%])  COWS (1 [4%])  DAST (1 [4%])  Dialysis adequacy (1 [4%])  Drugs craving (1 [4%])  HBI (1 [4%])  KOOS (1 [4%])  PCI revascularisation (1 [4%])  Penn alcohol craving scale (1 [4%])  Questionnaire of craving belief (1 [4%])  Rachmilewitz index (1 [4%])  Rating scale for heroin withdrawal symptoms (1 [4%])  Readiness to change ruler (1 [4%])  Relapse rate (1 [4%])  Response spread (1 [4%])  SADD (1 [4%])  SCCAI (1 [4%])  SCQ (1 [4%])  Self-efficacy for alcohol abstinence (1 [4%])  Severity of dependence (1 [4%])  Sexual function/activity (1 [4%])  SMAST (1 [4%])  SSS (1 [4%])  Stroke self-efficacy (1 [4%])  Substance specific craving scale (1 [4%])  Substance use recovery evaluator (1 [4%])  Substance used (1 [4%])  VAS drug cravings (1 [4%])  Withdrawal syndrome evaluation (1 [4%]) |
| **Adverse events (12 [48%])** | Adverse events (9 [36%])  Adverse effects (2 [8%])  Adverse cardiac events (1 [4%]) |
| **Cardiovascular performance (12 [48%])** | HR (8 [32%])  HRmax/peak (7 [28%])  HRV (3 [12%])  AF episodes (1 [4%])  Brachial artery FMD (1 [4%])  Calf blood flow (1 [4%])  Cardiac index (1 [4%])  Doppler ultrasound (1 [4%])  HR (deep breathing) (1 [4%])  Left atrial diameter (1 [4%])  Lower limb O_2_ delivery (1 [4%])  Peak exercise calf blood flow (1 [4%])  Time in AF (1 [4%]) |
| **Physical activity (11 [44%])** | Step count (8 [32%])  Accelerometry (3 [12%])  IPAQ (3 [12%])  ADL (3 [12%])  EE (2 [8%])  Exercise TFLB (2 [8%])  PA level (2 [8%])  AAP (1 [4%])  Adjusted activity score (1 [4%])  Baecke questionnaire (1 [4%])  CDAD-IADL (1 [4%])  Confidence during ADL (1 [4%])  Daily exercise (1 [4%])  Exercise beliefs and self-questionnaires (1 [4%])  Exercise intensity (1 [4%])  Frenchay activity index (1 [4%])  Home walking time (1 [4%])  Human activity profile (1 [4%])  Jebsen time (1 [4%])  Katz ADL (1 [4%])  Lawton ADL (1 [4%])  Moderate intensity activities (1 [4%])  NEADL (1 [4%])  OARS (1 [4%])  PA diary (1 [4%])  Minutes of PA/day (1 [4%])  PADS (1 [4%])  PASE (1 [4%])  PASIPD (1 [4%])  PASS (1 [4%])  SE-ADL (1 [4%])  SEES (1 [4%])  Self-reported walking (1 [4%])  YPAS (1 [4%]) |
| **Mobility/balance (11 [44%])** | TUG (9 [36%])  Berg balance scale (6 [24%])  One-leg stand (5 [20%])  Sit-and-reach (5 [20%])  Functional reach (4 [16%])  ABCS (3 [12%])  Four square step test (3 [12%])  Tinetti test (3 [12%])  Back scratch test (2 [8%])  Figure of 8 (2 [8%])  Postural sway (2 [8%])  Spinal ROM (2 [8%])  5-Cog dual task (1 [4%])  6-spot step test (1 [4%])  ACIF (1 [4%])  Balance confidence (1 [4%])  Balance platform (1 [4%])  BESTest (1 [4%])  Biodex balance (1 [4%])  Brunel balance assessment (1 [4%])  Centre of pressure confidence ellipse area (1 [4%])  Centre of pressure path length (1 [4%])  Contact time (1 [4%])  Double limb support (1 [4%])  Dynamic balance (1 [4%])  Dynamic postuography (1 [4%])  Elderly mobility scale (1 [4%])  FAB scale (1 [4%])  FAC (1 [4%])  FICSIT-4 (1 [4%])  Flamingo stand (1 [4%])  Fugl-Meyer assessment (1 [4%])  Gait cadence (1 [4%])  Gait economy (1 [4%])  Gait pattern (1 [4%])  Gait velocity (1 [4%])  Groningen-Meander walking test (1 [4%])  Harmonic ratio (1 [4%])  Hip/shoulder ROM (1 [4%])  IRGL (1 [4%])  Lateral flexion (1 [4%])  Lequesne index (1 [4%])  Life space assessment (1 [4%])  Limit of stability (1 [4%])  MCTSIB (1 [4%])  Mini-BESTest (1 [4%])  Modified physical performance test (1 [4%])  Physical self-maintenance scale (1 [4%])  Plantar pressure distribution (1 [4%])  POMA (1 [4%])  Postuography platform (1 [4%])  Postural ability (1 [4%])  PPA (1 [4%])  Reaction time (1 [4%])  RMI (1 [4%])  Southampton mobility assessment (1 [4%])  Spatio-temporal gait parameters (1 [4%])  Stance symmetry (1 [4%])  Step quick turn (1 [4%])  Stride cadence (1 [4%])  Stride frequency (1 [4%])  Stride length (1 [4%])  Tandem stance (1 [4%])  Tandem walk (1 [4%])  Timed balance test (1 [4%])  Tokya metropolitan institute of gerontology index of competence (1 [4%])  Walking ability questionnaire (1 [4%])  Walking work efficiency (1 [4%])  Wii balance (1 [4%]) |
| **Disability (9 [36%])** | Number of falls (4 [16%])  FIM (3 [12%])  Barthel index (2 [8%])  Sheehan disability (2 [8%])  Amputation (1 [4%])  Assessment of motor and process skills (1 [4%])  CDAD (1 [4%])  Community ambulation (1 [4%])  Distance until fatigue (1 [4%])  Fall rate (1 [4%])  Fall-related self-efficacy (1 [4%])  FAPS (1 [4%])  Fear of falling (1 [4%])  FFABQ (1 [4%])  FROP-Com (1 [4%])  LLFDI (1 [4%])  Modified falls efficacy scale (1 [4%])  Motor assessment scale (1 [4%])  Motor control (grip) (1 [4%])  Motricity index (1 [4%])  Number of fallers (1 [4%])  NOSGER (1 [4%])  ODI (1 [4%])  REPDS (1 [4%])  Roland-Morris disability questionnaire (1 [4%])  Support needs questionnaire (1 [4%])  Time to first fall (1 [4%])  Waddell disability index (1 [4%])  Walking/falls self-efficacy (1 [4%]) |
| **Blood pressure (8 [32%])** | SBP (5 [20%])  Blood pressure (4 [16%])  DBP (4 [16%])  Maximum blood pressure (1 [4%])  DBPpeak (1 [4%])  SBPpeak (1 [4%]) |
| **Physical function (8 [32%])** | SPPB (4 [16%])  Stair climb (3 [12%])  Chair rise (2 [8%])  PSFS (2 [8%])  Stride time (2 [8%])  Stride width (2 [8%])  5m max speed (1 [4%])  9-hole peg test (1 [4%])  AFI (1 [4%])  ARAT (1 [4%])  Ashworth scale (1 [4%])  Box and block test (1 [4%])  Cadence (1 [4%])  Chair stand test (1 [4%])  Community walking assessment (1 [4%])  Dual task performance (1 [4%])  Dual-task walking speed (1 [4%])  FSQ (1 [4%])  FTHUE (1 [4%])  Functional autonomy (1 [4%])  Functional fitness (1 [4%])  Functional walking questionnaire (1 [4%])  Gait endurance (1 [4%])  Gait speed (1 [4%])  Gait velocity (1 [4%])  GARS (1 [4%])  Hannover functional ability questionnaire (1 [4%])  Isometric quadricep endurance (1 [4%])  Jebsen hand test (1 [4%])  JOA (1 [4%])  Joint angles (1 [4%])  Lower muscle endurance (1 [4%])  Million index (1 [4%])  Number of squats in 1 minute (1 [4%])  RMA (1 [4%])  Self-perceived ambulation (1 [4%])  Self-reported functional status (1 [4%])  Senior fitness test (1 [4%])  Step length (1 [4%])  Step test (1 [4%])  Step time (1 [4%])  Step time variability (1 [4%])  Steps/min (1 [4%])  Stride length (1 [4%])  Trunk impairment scale (1 [4%])  Unspecified ‘gait’ (1 [4%])  Upper muscle endurance (1 [4%])  Upright motor control test (1 [4%])  VAS (1 [4%])  Walk ratio (1 [4%])  Walking economy (1 [4%])  Walking kinematic data (1 [4%])  Walking quality scale (1 [4%])  Walking ratios (1 [4%])  Walking speed (1 [4%])  WELCH (1 [4%])  WIQ (1 [4%]) |
| **Fatigue (8 [32%])** | FSS (4 [16%])  BFI (2 [8%])  FACT-F (2 [8%])  Fatigue assessment scale (2 [8%])  MFI (2 [8%])  PFS (2 [8%])  Borg RPE (1 [4%])  CIS (1 [4%])  CIS20r (1 [4%])  CRF (1 [4%])  ESAS (1 [4%])  FFMQ-SF (1 [4%])  FIS (1 [4%])  FQ (1 [4%])  FSMC (1 [4%])  Schwartz fatigue scale (1 [4%])  Unspecified ‘fatigue’ (1 [4%])  VAS (1 [4%]) |
| **Overall mental health (8 [32%])** | Global impression of change (2 [8%])  POMS (2 [8%])  RSES (2 [8%])  Self-efficacy scale (2 [8%])  11-point Likert scale for mood (1 [4%])  6-item mood disturbance (1 [4%])  CPCI (1 [4%])  CPSS (1 [4%])  Ewarts self-efficacy scale (1 [4%])  Feeling scale (1 [4%])  Felt arousal scale (1 [4%])  GHQ (1 [4%])  GHQ-20 (1 [4%])  GHQ-28 (1 [4%])  K6 (1 [4%])  London psychogeriatric rating scale (1 [4%])  MMPI (1 [4%])  Mood states (1 [4%])  NRS (1 [4%])  PANAS (1 [4%])  PANSS (1 [4%])  PTSD checklist (1 [4%])  Screening questionnaire of common mental disorders (1 [4%])  Self-esteem scale (1 [4%])  Situational confidence questionnaire (1 [4%])  Tennessee self-contempt scale (1 [4%])  Voids craving and negative mood scale (1 [4%]) |
| **Mortality (8 [32%])** | All-cause mortality (1 [4%])  100-day mortality (1 [4%])  180-day mortality (1 [4%])  Cardiovascular mortality (1 [4%])  Death (1 [4%])  Fatal/non-fatal MI (1 [4%])  Hospital stay mortality (1 [4%])  Long-term survival (1 [4%]) |
| **Pain (6 [24%])** | McGill pain (3 [12%])  NRS (3 [12%])  VAS (3 [12%])  BPI (2 [8%])  WHYMPI (2 [8%])  Aberdeen back pain (1 [4%])  Activity discomfort scale (1 [4%])  AIMS (1 [4%])  Back pain (1 [4%])  Borg RPE (1 [4%])  Bothersomeness (1 [4%])  Chronic pain status/intensity (1 [4%])  CPGS (1 [4%])  Dallas pain questionnaire (1 [4%])  ESAS (1 [4%])  FACT (1 [4%])  FAS (1 [4%])  FAST (1 [4%])  FFMQ-SF (1 [4%])  KOOS (1 [4%])  LBP rating scale (1 [4%])  Likert scale (1 [4%])  MOLBPQ (1 [4%])  MOS pain severity (1 [4%])  Number of days in pain (1 [4%])  OASI (1 [4%])  OMPSQ (1 [4%])  Pain behaviour (video analysis) (1 [4%])  Pain disability index (1 [4%])  Pain rating scale (1 [4%])  Perceived pain during exercise (1 [4%])  QBPDS (1 [4%])  Tender point count (1 [4%])  Total myalgic score (1 [4%])  Unspecified ‘pain’ (1 [4%])  Verbal rating scale (1 [4%])  WOMAC (1 [4%]) |
| **Lung function (5 [20%])** | FEV_1_ (2 [8%])  FVC (2 [8%])  PEF (2 [8%])  TLCO (2 [8%])  Unspecified ‘pulmonary/lung function’ (2 [8%])  FEV_1_/FVC (1 [4%])  FEV_1_/FVC % (1 [4%])  FEV_1_ % (1 [4%])  FVC % (1 [4%])  MVV (1 [4%]) |
| **Impact (5 [20%])** | SIP (3 [12%])  AIMS (1 [4%])  Carer burden index (1 [4%])  DAS-28 (1 [4%])  FIQ (1 [4%])  Health and social service use (1 [4%])  Illness intrusive questionnaire (1 [4%])  Illness perception questionnaire (1 [4%])  KOOS (1 [4%])  OASI (1 [4%])  Rheumatology attitude index (1 [4%])  RLOC (1 [4%])  SIS (1 [4%])  Zarit burden interview score (1 [4%]) |
| **Dyspnoea (4 [16%])** | Borg scale (3 [12%])  mMRC (3 [12%])  BDI (1 [4%])  UCSD SOBQ (1 [4%]) |
| **Sleep (4 [16%])** | PSQI (2 [8%])  Insomnia severity index (1 [4%])  NRS (1 [4%])  Sleep disturbance scale (1 [4%])  Sleep duration (1 [4%])  Sleep latency (1 [4%]) |
| **Hospitalisation (3 [12%])** | All-cause hospitalisation (2 [8%])  Admission rate (1 [4%])  Cardiovascular hospitalisation (1 [4%])  HF hospitalisation (1 [4%])  Hospital stay length (1 [4%]) |
| **Cognitive function (3 [12%])** | MoCA (2 [8%])  Stroop test (2 [8%])  Trail making test (2 [8%])  Paced addition serial attention task (1 [4%])  Pincus cognitive symptoms inventory (1 [4%])  Processing speed (1 [4%])  Rey auditory verbal learning test (1 [4%])  Wisconsin card sorting (1 [4%]) |
| **Adherence (3 [12%])** | Withdrawals (1 [4%])  Adherence (1 [4%])  Attendance (1 [4%]) |
| **Economic (3 [12%])** | % still on sick leave (1 [4%])  Costs/cost-effectiveness (1 [4%])  Degree of interference at work (1 [4%])  Healthcare utilisation (1 [4%])  Number of hours worked (1 [4%])  Number of work days (1 [4%])  QALY (1 [4%])  Return to work (1 [4%])  Sick leave days (1 [4%])  Sickness absence (1 [4%])  Sickness presentation (1 [4%])  Subjective working capacity (1 [4%])  Total length of leave (1 [4%])  Work ability (1 [4%])  Work ability index (1 [4%])  Work readiness (1 [4%]) |
| **Overall symptoms (2 [8%])** | SCL-90-R (2 [8%])  CGI (1 [4%])  Clinical outcome score (1 [4%])  General well-being after treatment (1 [4%])  Global assessment (1 [4%])  Global improvement (1 [4%])  Global perceived effect (1 [4%])  Global perceived outcome (1 [4%])  Global rating of change (1 [4%])  HSCL-25 (1 [4%])  Number improved (1 [4%])  Number symptom free (1 [4%])  Overall perceived benefit from treatment (1 [4%])  PROMIS-29 (1 [4%])  Reported improvement/worsening in specific positions (1 [4%])  Response to treatment (1 [4%])  Self-reported recovery (1 [4%]) |
| **Exacerbation (1 [4%])** | Exacerbation frequency (1 [4%]) |
